# Silk Polymers and Nanoparticles: A Powerful Combination for the Design of Versatile Biomaterials

**DOI:** 10.3389/fchem.2020.604398

**Published:** 2020-12-01

**Authors:** Cristina Belda Marín, Vincent Fitzpatrick, David L. Kaplan, Jessem Landoulsi, Erwann Guénin, Christophe Egles

**Affiliations:** ^1^Laboratory of Integrated Transformations of Renewable Matter (TIMR), Université de Technologie de Compiègne, ESCOM, Compiègne, France; ^2^Laboratoire de réactivité de surface (UMR CNRS 7197), Sorbonne Université, Paris, France; ^3^Department of Biomedical Engineering, Tufts University, Medford, MA, United States; ^4^Biomechanics and Bioengineering, CNRS, Université de Technologie de Compiègne, Compiègne, France

**Keywords:** natural polymers, silk, nanoparticles, bioactive biomaterials, regenerative medicine

## Abstract

Silk fibroin (SF) is a natural protein largely used in the textile industry but also in biomedicine, catalysis, and other materials applications. SF is biocompatible, biodegradable, and possesses high tensile strength. Moreover, it is a versatile compound that can be formed into different materials at the macro, micro- and nano-scales, such as nanofibers, nanoparticles, hydrogels, microspheres, and other formats. Silk can be further integrated into emerging and promising additive manufacturing techniques like bioprinting, stereolithography or digital light processing 3D printing. As such, the development of methodologies for the functionalization of silk materials provide added value. Inorganic nanoparticles (INPs) have interesting and unexpected properties differing from bulk materials. These properties include better catalysis efficiency (better surface/volume ratio and consequently decreased quantify of catalyst), antibacterial activity, fluorescence properties, and UV-radiation protection or superparamagnetic behavior depending on the metal used. Given the promising results and performance of INPs, their use in many different procedures has been growing. Therefore, combining the useful properties of silk fibroin materials with those from INPs is increasingly relevant in many applications. Two main methodologies have been used in the literature to form silk-based bionanocomposites: *in situ* synthesis of INPs in silk materials, or the addition of preformed INPs to silk materials. This work presents an overview of current silk nanocomposites developed by these two main methodologies. An evaluation of overall INP characteristics and their distribution within the material is presented for each approach. Finally, an outlook is provided about the potential applications of these resultant nanocomposite materials.

## Introduction

Silk is a natural polymer originating from various insect and spider species. It is composed of two different proteins, sericin and fibroin, among which fibroin is an FDA-approved material for some medical devices. Due to the remarkable mechanical properties, biocompatibility and biodegradability, fibroin has been shaped into various scaffolds, including sponges, electrospun mats, microspheres, hydrogels, aerogels, foams, and 3D printed structures. These silk-based scaffolds are particularly investigated in various tissue engineering applications, including for bone, nerve, skin, cartilage or corneal regeneration, but also as vehicles for drug delivery. These materials are also studied in other scientific areas such as for pollution control, electronics, optics, and material science in general.

Moreover, silk fibroin can be blended with different additives to form scaffolds with new properties. Among these, nanocomposites which comprise silk polymers and nanoparticles (NPs) have gained increasing interest due to the outstanding properties of the NPs, which differ in their properties from bulk materials. Accordingly, the development of silk-NPs nanocomposites has triggered growing interest for both academic and industrial research. Combining the assets of silk fibroin materials with those from NPs is appealing to obtain new properties that are unattainable by “classical” composites consisting of the same bulk materials.

In this review, we present an overview of current nanocomposites constituted of silk fibroin and NPs with particular attention to inorganic NPs (INPs), typically metals, oxides, and bioceramics. First, we present current knowledge on silk-based scaffolds and their main applications. We then describe the principal INPs (synthesis, properties) used to produce these composites. Finally, we provide an outlook of the potential applications of the resultant nanocomposite materials and guidelines for tuning their properties and functions.

## Silk

### Silk Structure and Extraction

Silks are protein biopolymers produced by many members of the arthropod family such as spiders, silkworms, flies, and silverfish. Each arthropod produces silk components with a different amino acid composition, resulting in different structural properties (Xiong et al., 2018). Mechanical properties are different, with some spider silks being stronger than silkworms silk. In addition, silk properties are influenced by other parameters, such as the environment and arthropod nutrition, giving rise to different silk types produced by the same species (Koh et al., 2015). Among existing silks, mulberry worm silks are the most commonly used for textiles and biomedical applications. Although some spider silks have greater tensile strength, toughness and extensibility, the cannibalistic nature of spiders makes the development of industrial production of spider silks with high yield impossible. *Bombyx mori* silkworms, on the other hand, were domesticated for industrial silk production centuries ago. As such, silkworm silk is almost exclusively used for medical and related applications, and for this reason only *B. mori* silk is considered in the following sections.

#### *Bombyx mori* Silk Structure

Silk fibers consist of two main proteins form *B. mori* silk: fibroin and sericin. Silk fibers are composed of fibroin microfibrils assembled into filaments. Silk fibers consist of two fibroin filaments each produced by one of the worm's salivary glands during spinning. Both filaments are then covered by sericin, an adhesive and hydrophilic protein to form the structural unit (Poza et al., 2002).

Fibroin is an hydrophobic protein formed by two chains: a light chain (L-chain, ~26 kDa); and a heavy chain (H-chain, ~390 kDa). The two fibroin chains are covalently linked by a disulfide bond between two cysteines, forming a H-L complex. The formation of this complex is essential for the secretion of silk fibroin from producing cells to the glands. The primary structure of silk fibroin (SF) is formed by highly repetitive sequences composed mainly of glycine (43%), alanine (30%) and serine (12%). Other amino acids such as tyrosine (5%), valine (2%), and tryptophan are present in smaller proportions (Koh et al., 2015). The primary structure of the H-chain contains 12 repetitive hydrophobic domains interspersed with 11 non-repetitive hydrophilic regions. Three different polymorphs of SF (silk I, II, and III) have been reported; Silk I adopts a coiled structure and is found in the silk stored in the arthropods' glands. This conformation is also found in regenerated aqueous dispersions *in vitro*. Silk II corresponds to the antiparallel β-sheet crystal structure obtained once silk has been spun. In the laboratory, this polymorph results from the exposure of silk I to mechanical/physical and chemical treatments, such as stirring, heating, exposure to methanol or water annealing procedures. The formation of the β-sheet structure is possible due to the rearrangement of the repetitive regions that form the H-chain of SF, and the intra and intermolecular interactions by hydrogen bonding, van Der Waals forces and hydrophobic interactions. X-ray diffraction (XRD) analysis of the crystallinity regions of SF found an antiparallel β-sheet structure. The non-repetitive domains adopt a coiled conformation. Silk II excludes water from the structure, giving strength to the protein filament and making it insoluble in water and other solvents like mild acids or bases. The third polymorph, silk III, adopts a helical structure at air-water interfaces (Vepari and Kaplan, 2007).

#### Regenerated Silk Fibroin Extraction

Some studies have shown that sericin may induce an immunogenic response in the human body while SF has been approved by the FDA for medical use in the US (Zhang et al., 2017). Sericin is therefore removed from silk for biomedical applications (Rockwood et al., 2011). *B. mori* silk cocoons are processed to obtain a regenerated SF solution. This procedure differs from that used by the textile industry as the final objective is not to obtain silk fibers but a SF solution. The goal is to bring silk (polymorphs II and III) to the initial state found in the glands of the worm (silk I) before being spun. This transformation can be achieved by denaturing SF proteins, which result in protein solution (Rockwood et al., 2011). Briefly, silk cocoons are boiled in a sodium carbonate (Na_2_CO_3_) solution to remove the sericin (soluble in hot water) that glues together the SF filaments. Boiling time is a crucial parameter influencing the properties of SF in solution. Longer times will disrupt SF chains to lower molecular weight. Boiling silk cocoons for 30 min will result in ~100 kDa fibroin proteins (Rockwood et al., 2011). Once boiled, the resulting entangled cotton-like fibers are abundantly rinsed in distilled water (to remove any remaining sericin) to obtain the SF dispersion prior to solubilization in various, albeit limited, salt and related systems.

Different solvents can be used for complete dissolution of the SF, the most common being lithium bromide (LiBr) (Xiong et al., 2018). Briefly, a LiBr solution is mixed with the extracted and dried SF fibers and heated for a specific time (Rockwood et al., 2011). LiBr allows the destabilization of hydrogen bonds found in silk II polymorph; allowing the shift to the silk I structure (Xiong et al., 2018). LiBr is a chemical hazard that can cause skin and eye irritation, encouraging the search for alternative solutions. Another solvent used to dissolve silk fibroin fibers is a ternary system composed of calcium chloride, ethanol and water (Song et al., 2017). Ionic liquids have also been used to dissolve silk, such as 1-butyl-3-methylimidazolium chloride (BMIM Cl), 1-butyl-2,3-dimethylimidazolium chloride (DMBIM Cl) and 1-ethyl-3-methylimidazolium chloride (EMIM Cl) (Phillips et al., 2004). The solution obtained in the above process must be dialyzed with distilled water to remove the salts. Finally the protein solution is centrifuged twice to remove any solid impurities (Rockwood et al., 2011). The resultant solution is around 6–8% w/v SF and can be further concentrated up to around 30%. Regenerated SF dispersion should be handled with care, as many procedures such as heating, stirring or pH variations will induce protein rearrangement, forming β-sheet structures and resulting in the gelation of the solution. Because of this, regenerated SF dispersion should be stored at 4°C and for no longer than 1 month.

Unlimited storage can be achieved by lyophilizing the SF solution. Lyophilized product can be redissolved in water, formic acid or 1,1,1,3,3,3-hexafluoro-2-propanol (HFIP) (Rockwood et al., 2011) at the desired concentration.

### Silk-Based Materials

**Figure 1:**
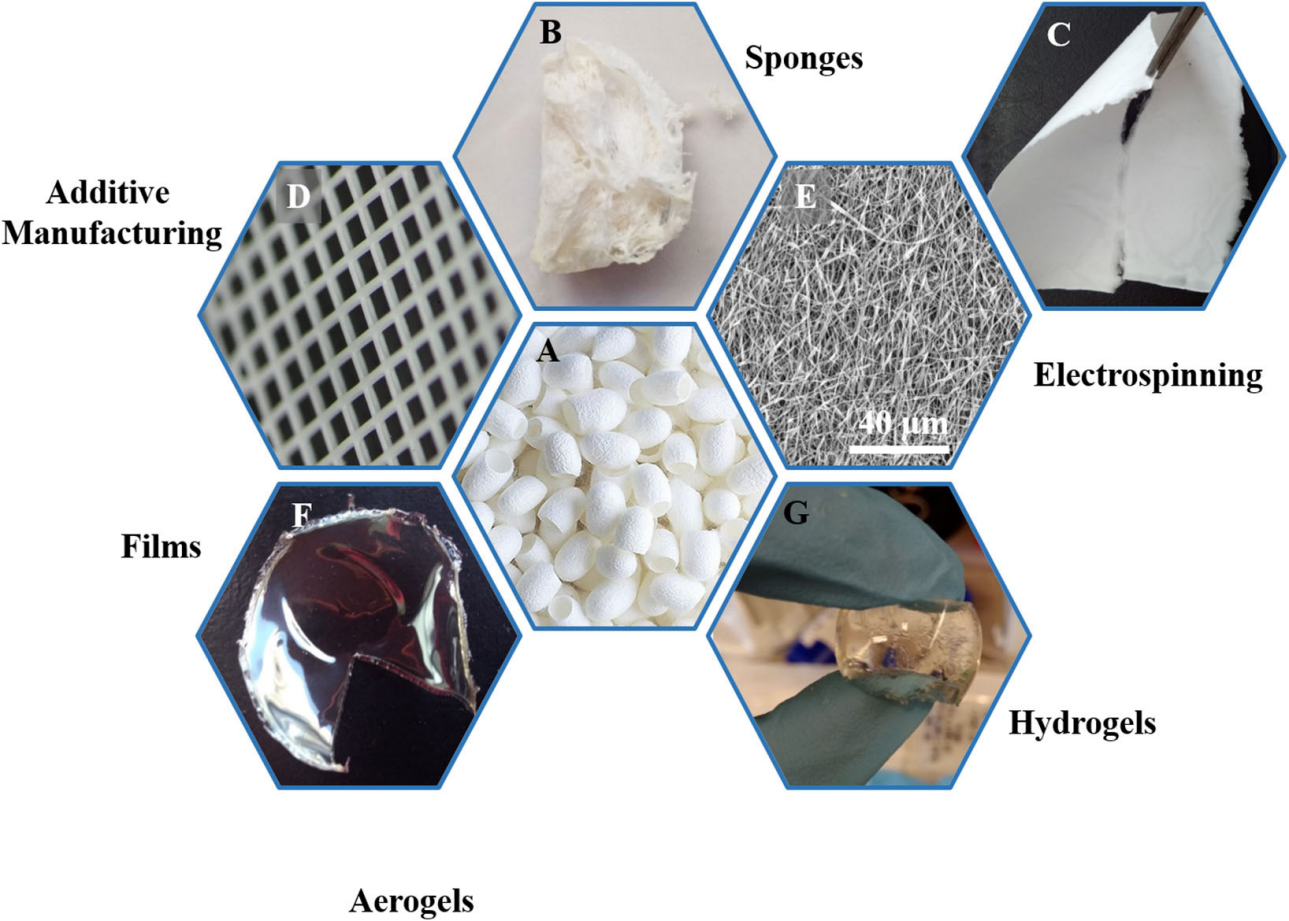
Overview of the various biomaterials obtained from of silk fibroin extracted from silk cocoons **(A)**. Silk cocoons **(A)**, sponges **(B)**, macroscopic and microscopic images of electrospun mats **(C,E)**, 3D printed structures **(D)**, films **(F)**, hydrogels **(G)**, and aerogels **(H)**.

Silk is traditionally known for its wide use in the textile industry given its lightweight, soft touch, and luster. The β-sheet structure found in silk II polymorph is responsible for silk's mechanical properties, which are well-beyond most known biopolymers. Because of its unique properties and its versatility, a wide range of materials with various properties can be obtained from a SF dispersion or its lyophilized powder. Figure 1 shows some of the multiple materials that can be obtained from silk fibroin. Most techniques used to construct SF materials are based on the controlled formation of β-sheet structures. Which enables tailoring mechanical properties, rates of biodegradation, and the degree of solvent dissolution of the silk.

#### Silk Fibroin Sponges

Silk fibroin sponges are 3D porous materials for which pore size and interconnectivity can be controlled depending on the production method. Silk fibroin sponges can be produced by mixing the silk solution with a porogen (e.g., salt or sugar crystals, polymer or mineral beads) and subsequently inducing silk gelation. Many different procedures have been described, such as the use of sodium chloride (salt leaching), freeze casting (Cai et al., 2017a), or HFIP solvent (Rockwood et al., 2011). Silk fibroin sponges can be used as scaffolds for bone tissue regeneration due to their macroporous structure that can be tailored to promote the enhanced formation of new and vascularized bone tissue (Karageorgiou and Kaplan, 2005). Several *in vitro* and *in vivo* studies have demonstrated the potential of cellularized scaffolds or acellular silk materials for bone regeneration (Bhattacharjee et al., 2017).

#### Electrospun Mats

Electrospinning is a simple technique that consists of using of an electric field to spin a polymer solution into a non-woven mat composed of nanometer diameter fibers. During electrospinning, the polymer solution is placed in a syringe with a conductive needle connected to a high voltage electric field (5–40 kV). A grounded conductive collector is placed at a distance in front of the needle. While the polymer solution is extruded through the needle, the high voltage electric field induces its stretching, allowing the formation of nanofibers. During the process, the solvent evaporates at rates dependent on its intrinsic properties and experimental conditions, and the fibers are deposited on the collector due to the voltage difference (Rockwood et al., 2011). The nature of the collector used impacts the fiber alignment. If a flat static collector is used, fibers are randomly deposited. A rotating mandrel used as a collector results in alignment of the deposited fibers (Rockwood et al., 2011), useful for some applications, such as direction control of growth in neural regeneration (Belanger et al., 2018).

The fiber diameter obtained by electrospinning can be modulated by adjusting extrinsic parameters: polymer concentration, solvent, polymer extrusion flow rate, needle-to-collector distance, and the applied voltage. The electrospinning procedure may also be sensitive to intrinsic factors such as the molecular weight of the silk. Silk fibroin electrospinning is also dependent on humidity and temperature. A non-controlled variation in one of these parameters will alter the characteristics of final material. Electrospun mats can be used in the field of wound dressings, textiles, wearable electrodes, nerve guides, and other systems (Yukseloglu et al., 2015).

#### Microspheres

Silk microspheres can be produced by several methods; encapsulation in fatty acids creating an emulsion, phase separation of silk from another polymer such as poly(vinyl alcohol) (PVA) (Rockwood et al., 2011), or adding potassium phosphate to the aqueous silk solution (Lammel et al., 2010). Silk has been extensively used for drug delivery, both as a vehicle and due to its stabilizing effect on bioactive molecules and enzymes (Li A. B. et al., 2015). Silk microspheres are of interest as an encapsulating material in this field, because modulating their degradation rate results in controlled release of the contents (Lan et al., 2014; Li H. et al., 2017).

#### Hydrogels

Hydrogels are of interest due to their mechanical properties akin to soft tissues in the body. In addition, their capacity to swell and retain a high liquid volume renders them interesting for depollution applications, such as in environmental hazard removal (Hou et al., 2018). Hydrogels can be used to replace damaged soft tissues such as cartilage, the intervertebral disc, cornea and skin, among others. SF hydrogels can be found in applications such as drug delivery (Niu et al., 2019), tissue engineering (Vidal et al., 2019), regenerative medicine (Fernández-García et al., 2016; Frauchiger et al., 2017; Li et al., 2020), and catalysis (Luo and Shao, 2017).

The formation of hydrogels consists of the rearrangement of SF molecules to form crystalline structures as physical crosslinks. For this purpose, many protocols have been described in the literature to control the characteristics of the gels. Physical-, photochemical, and chemical-induced gelation methods have been reported. “Physical gelation” occurs through the formation of physical interactions, including electrostatic (coulombic) and van der Waals. Physical gelation protocols include solution sonication (Fernández-García et al., 2016), vortexing, the application of an electrical current or a pH decrease below the pI (Rockwood et al., 2011). “Chemical gelation” consists of the formation of new covalent bonds (crosslinks) via enzymes, chemical catalysts, or other chemical species. Protocols mostly involve the use of enzymes, such as oxidases, phosphatases, transglutaminases, or peroxidases (Nezhad-Mokhtari et al., 2019).

##### Enzyme-assisted crosslinking

Enzymatic crosslinking offers *in situ* crosslinking and interactions with the surrounding extracellular matrix (Nezhad-Mokhtari et al., 2019). Moreover, enzymatic-catalyzed reactions are specific and their rates are tunable, thus allowing a good control over the reaction products. Although many enzymes have been used to form such hydrogels, horseradish peroxidase (HRP) is the most extensively used.

##### HRP-crosslinked hydrogels

Commercially available horseradish peroxidase is extracted from the roots of *Armoracia rusticana*. In the plant, many different isoenzymes have been identified, although the most common one is HRP C. HRP is an oxidoreductase that catalyzes the conjugation of phenol and aniline derivatives in the presence of hydrogen peroxide (H_2_O_2_). In proteins, the action of HRP results in the formation of dityrosine bonds between the phenol groups present on tyrosine. The incorporation of tyramine in enables crosslinking by HRP (Bang et al., 2017; Bi et al., 2019). The use of this enzyme to crosslink peptides, polysaccharides and polymers has been extensively described in the literature (Hoang Thi et al., 2019; van Loo et al., 2020). In particular, HRP-crosslinked SF hydrogels have been described (Partlow et al., 2014), including the structure, crosslinking kinetics, rheological, and mechanical properties, as well as the cytotoxicity and biocompatibility of the hydrogels formed in the process. The properties of these SF hydrogels were tunable depending on several parameters, including silk fibroin molecular weight and concentration. In addition, the all-aqueous procedure, together with its biocompatibility and *in vivo* tolerance, makes this hydrogel a good candidate for biomedical applications and the encapsulation of biological factors (growth factors, hormones, cytokines), while preserving their activity.

#### Aerogels

Aerogels are open porous materials of very low density that derive from replacing the liquid component of a gel by a gas. Silk aerogels are generally produced by freeze drying a silk solution or hydrogel. Similar to sponge materials, a 3D porous scaffold is obtained (Xiong et al., 2018) and pore size and distribution can be tuned by controlling the rate of freezing. One example of the process is an ice templating technique that consists in controlling ice crystal growth in the silk sample to obtain a desired structure. Microchannel containing silk scaffolds can be obtained by this technique (Qian and Zhang, 2011; Xiong et al., 2018). Aerogels have been used as fire retardant materials, thermal insulators (Maleki and Huesing, 2019), pollution control materials, and as biomaterials (Mandal and Kundu, 2009).

#### 3D Printed Structures

The development of 3D printing technologies has made it possible to print polymer dispersions, including silk structures. This approach controls the shape and dimensions of the structures (Mu et al., 2020). Many silk-containing bioinks or 3D printing techniques are being developed with silk inks. Some approaches have focused on the properties of silk to obtain a construct, for example, by printing in a saline bath to induce hierarchical assembly of the silk proteins (Mu et al., 2020), or using freeform printing in a bath of synthetic nanoclay and polyethylene glycol (PEG) for a one-step process of printing and *in situ* physical gelation (Rodriguez et al., 2018). Other strategies have focused on mixing SF with other polymers and thickening agents, such as PEG (Zheng et al., 2018), polyols (Jose et al., 2015) or the polysaccharide Konjac glucomannan (Sommer et al., 2016). 3D printed structures are of interest in the tissue engineering field, as they allow the manufacturing of complex and patient-tailored shapes with controlled macroporosity.

#### Silk Foams

Silk memory foams offer a promising and minimally invasive solution for soft tissue regeneration. These materials can be compressed prior to implantation, and then have the ability to recover their volume post-injection or implantation (Brown et al., 2017). *In vivo*, these materials have shown promise as soft tissue fillers, colonized by migrating cells and integrating with the surrounding native tissue (Bellas et al., 2015). These foams can be used as a drug delivery vehicles for bioactive molecules, and their degradation has been tuned using pre-loaded enzymes (Chambre et al., 2020). Likewise, these foams have been loaded with Dinutuximab, a monoclonal antibody with clinical potential for patients with high-risk neuroblastoma (Ornell et al., 2020). These silk foams induced a significant decrease in tumor growth rate in a mouse orthotopic tumor model. Overall, these materials are extremely well-suited for soft tissue regeneration and localized drug-delivery at an injury site.

#### Microneedles

The mechanical properties, biocompatibility, biodegradability, benign processing conditions, and stabilizing effect of silk on biological compounds has made it a important candidate for the fabrication of microneedle systems for drug delivery. The degradation rate of SF and the diffusion rate of the entrained molecules can be controlled by adjusting post-processing conditions (Yin et al., 2018). The microneedles can be further combined with other materials to make composite microneedles and further tune the drug release profile. These microneedles have been prepared with insulin (Wang S. et al., 2019), antibiotics (Tsioris et al., 2012), and vaccines (Demuth et al., 2014; Boopathy et al., 2019). Products based on this technology are currently being developed and commercialized for therapeutic applications.

#### Hard Silk Materials

The mechanical properties of regenerated silk materials can be tuned for orthopedic applications requiring hard materials by controlling the fabrication process (Li et al., 2016). Bulk regenerated silk-based materials with excellent mechanical properties were generated through a biomimetic, all-aqueous process. These materials replicated the nano-scale structure of natural silk fibers and demonstrated excellent machinability, allowing the fabrication of resorbable bone screws, intermedullary nails and fixation plates. These devices allowed functionalization with bioactive molecules like antibiotics, morphogens, or micro RNAs (James et al., 2019). Recent work developed a thermal processing method allowing the direct solid-state molding of regenerated silk nanoparticles into bulk “parts” or devices with tunable mechanical properties (Guo et al., 2020). Thus, robust materials that retain biocompatibility, degradability and machinability, without the time, cost, and stability limitations of using silk solution-derived methods represents a significant advance.

#### Films

Silk can be processed into thin films by air drying, methanol- or water-annealing, or even electrogelation (Stinson et al., 2020). Glycerol can be added to the formulation to obtain flexible silk films (Lu et al., 2010). While silk films are promising in the field of drug delivery (Zhou et al., 2014), or for the long-term stabilization of vaccines (Stinson et al., 2020), they also have direct applications in tissue engineering. Their interesting optical transparency and thin format make them candidates for corneal tissue models. The films sustain cell adhesion and growth; they can also be physically and chemically patterned to mimic the cellular and ECM organization of the cornea (Lawrence et al., 2009). Pores can also be added in the films to enhance trans-lamellar nutrient diffusion and cell-cell interactions. These films can be further stacked into multi-lamellar, helicoidal structures, and functionalized with RGD-peptides, allowing a biomimetic 3D corneal model (Gil et al., 2010).

## Applications

The versatility of silk materials and their tunable properties drive interest for many applications, particularly in tissue engineering (Kundu et al., 2013; Li G. et al., 2015), wearable electronics (Kim et al., 2010), and pollution control (Ling et al., 2017; Gao et al., 2018).

### Silk Fibroin Biocompatibility

Evaluating the biocompatibility of any medical device is a crucial step during its development. Biocompatibility is the fact that a biomaterial is “accepted” by the organism with minimal cytotoxicity and immunogenicity with the aim to prevent any rejection of the biomaterial. However, biocompatibility is dependent on the shape and the time of contact and use of the material. Therefore, every material should be tested in their final form to assess their compatibility. Silk materials have long been used as silk sutures; however, hypersensitivity has been shown in some cases, even it remains scarce. Silk being a foreign body material, when implanted, a mild inflammatory response is generally observed. This immunogenic response has been linked to the presence of sericin (residual or due to the use of non-degummed silk) within the material (Kundu et al., 2013). However, no immunogenicity has been found when using materials composed of a single silk protein, either fibroin or sericin (Bandyopadhyay et al., 2019). Many studies have shown the biocompatibility of alkali heat degummed silk materials, such as SF electrospun mats, films, gels, and microparticles. Interestingly, *in vivo* studies showed that silk induced a lower inflammatory response compared to type I collagen and PLA (Rockwood et al., 2011). Finally, the FDA has approved the use of silk for medical uses in the US (Zhang et al., 2017).

### Biomedical Applications

Silk materials can match most of the challenges in the biomedical field due to their mechanical robustness, biocompatibility, and biodegradability. The various material formats obtained from silk, as described above, can be used in many different medical applications.

Silk sutures have been used in the medical field for a long time, and were patented in 1966, thus establishing the possibility to use silk in medicine. Silk sutures were first developed to overcome the mechanical problems encountered with traditional sutures. Surgical sutures require a great tensile strength to keep both ends of the wound tight together even under physiological movements such as heartbeat, stomach or intestinal peristalsis, or muscle contraction and relaxation. In addition, surgeons should be able to knot sutures. Silk was a suitable candidate fiber as it met all these requirements. Since then, silk materials have been developed for many applications in the biomedical field, such as for wound dressings, skin, bone, cartilage, ocular, vascular, neuronal, and tissue regeneration in general (Kundu et al., 2013).

#### Wound Dressing

After sutures, wound dressings are probably the most common application of silk materials in the biomedical field. Several studies have found that silk materials induce faster reepithelization than conventional materials in skin burn wounds. Functionalization of electrospun silk materials has been pursued, such as with epidermal growth factor (EGF) and silver sulfadiazine, improving the overall wound healing process (Gil et al., 2013). Functionalization was also be achieved using silk microparticles (Li X. et al., 2017), such as with insulin for chronic wound healing applications. Insulin was chosen because of its contribution to wound healing and its acceleration of reepithelization. The overall wound healing effect was studied *in vivo* in diabetic rats and the insulin-loaded SF materials resulted in increased wound closure rates compared to non-loaded SF materials and conventional gauze.

#### Skin Equivalents

Silk-based materials have emerged in the field of skin equivalents due to the ability to improve wound closure, as well as its biocompatibility and biodegradability properties. One model of artificial skin was produced by silk electrospinning (Sheikh et al., 2015). Three different electrospinning techniques were compared: (i) traditional electrospinning (TE), (ii) salt leaching electrospinning (SLE), and (iii) cold plate electrospinning (CPE). CPE materials provided thicker materials, as ice crystals preserved the conductivity of the deposited material, thus enhancing the deposition of polymer depth. In addition, CPE-obtained materials showed increased cell infiltration and the possibility to generate an artificial skin substitute when culturing keratinocytes at the air-liquid interface. Finally, CPE can be used over curved surfaces, thus, making it easier to produce personalized skin systems.

Cells were included in a silk-based skin equivalent (Vidal et al., 2019). This complex skin equivalent included adipose tissue, endothelial cells, keratinocytes, neural cells, immune cells, and vascularization systems. The hypodermis was constructed on a silk sponge by salt leaching. Dermis and epidermis layers were shaped into a hydrogel containing complete cell culture media, silk, collagen, and fibroblasts. The two layers of materials were then combined to form a full-thickness skin equivalent. The presence of silk in the material was crucial to overcome the concerns about collagen skin equivalents, which is construct contraction over time; this is prevented by the silk due to its stable structure. In addition, the mechanical properties of silk-containing hydrogels were closer to skin than pure collagen materials. Moreover, silk-containing materials were useful for up to 6 weeks *in vitro*, providing the possibility to study patient-specific immune and neuronal responses for a longer period of time.

#### Bone Regeneration

Bone tissue engineering materials have been described (Bhattacharjee et al., 2017) with characteristics including: mechanical properties comparable to bone, biocompatibility, bioresorption and the capacity to deliver osteoprogenitor cells and growth factors. In addition, scaffolds should be able to provide mechanical integrity until the bone is completely regenerated, as they have to support high mechanical loads and stimuli. Resorbable materials are preferred as they avoid the need for a second surgery to remove the devices or implant. Collagen is the preferred material; however, a lack of mechanical properties limits its utility, synthetic polymers are used as alternatives. An extended review on silk-based materials for bone tissue engineering has been published (Bhattacharjee et al., 2017), and highlights the potential to exploit the versatility of silk materials as cellularized scaffolds or acellular biomaterials for bone tissue engineering. For example, guided bone regeneration was successfully achieved with silk membranes (Cai et al., 2017a). The main objective was to generate a material able to avoid connective tissue invasion into a bone defect after surgery. Invasion of the defect by soft tissue makes bone regeneration impossible, resulting in the local loss of function. In their *in vivo* experiments, the material performed as well as commercially available products.

#### Vascular Tissue Engineering

The gold standard for cardiovascular disease is autologous transplantation (autografts). However, limited donor tissue availability as well as donor site morbidity drive the need to develop new materials and tissue engineering approaches. Some synthetic materials are already used to construct vascular grafts, namely expanded polytetratfluoroethylene (ePTFE, Teflon®) and polyethylene terephthalate (PET, Dacron®). These materials are largely used for large diameter vessels or artery replacement. However, when replacing small diameter vessels (<6 mm) these materials fail when compared with autografts. Moreover, clinical complications such as aneurysms, intimal hyperplasia and thrombosis are associated with their use. Due to their characteristic mechanical properties and biocompatibility, silk materials have also been used in the field of vascular tissue engineering. Small vessel graft were developed with silk, where the diameter of the tube could be tailored and the graft was rich in β-sheet-structure to confer appropriate mechanical properties (Lovett et al., 2007). The incorporation of polyethylene oxide into the silk dispersion resulted in optimal porosity once the PEO was removed, enabling small protein transport but limiting endothelial cell migration. Vessel grafts were produced by combining two electrospun layers and an intermediate textile layer (Alessandrino et al., 2019). The mechanical properties were similar to native arteries, had biocompatibility, cell adhesion and blood hemocompatibility (no complement activation). However, further optimization is needed to reduce a foreign body response *in vivo* in sheep and minipigs.

#### Nerve Regeneration

Neural guidance is a key factor for efficient nerve regeneration. A three-layered silk electrospun material (aligned-random-aligned fibers) was generated to address this need (Belanger et al., 2018). The aligned electrospun silk material induced alignment in Schwann cells in contrast to the random growth found when cultured on glass coverslips. *In vivo* experiments in rats showed successful nerve regeneration after 4 months, with mechanical properties matching the tensile stress of the rat sciatic nerve (ca. 2.6 MPa). The insulating characteristics of silk materials may impair electrical potential for neural action and communication, thus composite approaches may be useful.

#### Drug Delivery

The controllable degradation rate of SF materials in the body enables their use as drug delivery devices. Gentamicin sulfate impregnated gelatin microspheres were embedded into silk scaffolds obtained by freeze drying (Lan et al., 2014). The material showed a reduced inflammatory response and accelerated reepithelization *in vivo* while exhibiting antibacterial properties. Similarly, dual drug-loaded silk materials were developed (Li H. et al., 2017). Silk microspheres containing curcumin were prepared and blended into a silk dispersion with the drug doxorubicin hydrochloride (DOX-HCl). By electrospinning, nanofiber silk materials were obtained containing the hydrophilic DOX-HCl in the shell and the hydrophobic curcumin in the core. Silk hydrogels can also be used for drug delivery. Silk hydrogels were loaded with bevacizumab, an anti-vascular endothelial growth factor antagonist (Lovett et al., 2015). Bevacizumab is a therapeutic agent for the treatment of age-related macular degeneration, an eye disease characterized by the progressive loss of vision. The intravitreal injection of hydrogels in rabbits' eyes showed sustained drug release for up to 90 days.

### Other Applications

Although silk is increasingly being developed in the biomedical field, interesting applications have also emerged in other areas, such as for pollution control (Ling et al., 2017; Gao et al., 2018), electronics (Kim et al., 2010), thermal insulators, and fire retardants (Maleki et al., 2018). For example, silk adsorbs many chemicals and metals, which renders it interesting for water and air pollution. With this objective, electrospinning has been used to develop silk air filters (Gao et al., 2018) that have high efficiency air filtration (up to 99.99%) for particles with mean diameters from 0.3 to 10 μm, and a decreased pressure drop in comparison with state-of-the-art materials. In addition, silk filters are biodegradable and can be involved in recycling or sustainability processes.

## Nano-Objects

Nano-objects are objects with at least one of their three dimensions at the nanoscale (smaller than 100 nm). Nano-objects are classified into three groups: nanoplates, nanofibers, and nanoparticles (NPs). According to the IUPAC, NPs are “particles” of any shape with dimensions in the 1–100 nm range. These nano-objects exhibit a high surface-to-volume ratio that is particularly useful in many fields, such as in catalysis and sensing. In addition, unexpected and tunable properties appear at the nanoscale, differing from the bulk material.

NPs are rapidly coated by biomolecules, mostly proteins, when injected into biological fluids, leading to the formation of a “biomolecular corona” (Monopoli et al., 2012). The stability of NPs in solution depends on the equilibrium of attractive and repulsive forces between NPs, influenced by physicochemical conditions including the ionic strength, nature of the ions, pH, temperature, and the presence of bio-organic compounds (e.g., steric effects). Destabilization of the NPs solution may result in aggregation and precipitation. Given these considerations (protein corona formation and stability of NPs solution) it is unlikely that NPs preserve their initial size over time in the body. Large-sized NPs can be easily eliminated from the body through conventional routes. The remaining NPs can be taken up, stored and even degraded by cells to limit bioavailability (Van de Walle et al., 2019; Balfourier et al., 2020; Plan Sangnier et al., 2020).

NPs can be synthesized by top-down or bottom-up methods. Top-down synthesis consists of breaking down the bulk material until the obtention of nanosized particles, such as ball-milling, laser ablation, and lithography. Bottom-up synthesis is performed by building up the nanomaterial atom by atom or molecule by molecule. Bottom-up methods are liquid synthesis and include chemical precipitation, sol-gel processes and micellar and inverse micellar synthesis, hydro/solvo-thermal methods, etc. (Su and Chang, 2017). Many different synthesis routes are actually used for different materials as a standard and consistent synthesis method for all NPs has not yet been found to our knowledge.

### Noble Metal Nanoparticles

Noble metal NPs are of particular interest in materials because the reduction of material needed allows decreasing costs and lower its environmental impact. Many noble metal NPs are currently used in several applications such as catalysis, biomedicine, environment depollution or electronics.

#### Gold NPs

Gold NPs are probably the most well-known type of NPs since the preparation of the colloidal “ruby” gold by Michael Faraday in the 19th century. These NPs are used in many fields, from medicine (imaging, diagnostics, therapeutics) to electronics, essentially due to their unique reactivity and optical properties emerging only at the nanoscale.

Gold NPs have been used for their surface plasmon resonance (SPR) effect that results in an enhancement of the electromagnetic field in the surface of the NPs and optical extinction at the plasmon resonance frequency. SPR allows gold NPs to be used as hyperthermia agents due to their capability to absorb light at a given frequency and transforming the energy into heat (Plan Sangnier et al., 2019; Balfourier et al., 2020). Gold NPs have also been largely used in sensing systems (Zhou et al., 2015; De Crozals et al., 2016; Szekeres and Kneipp, 2019), as contrast agents for computed tomography (CT), as antibacterial agents (Cui et al., 2012), and as catalyst (Alshammari, 2019; Begum et al., 2019).

#### Silver NPs

Silver is known for its antibacterial action. The use of silver as an antibacterial agent had decreased with the arrival of antibiotics. However, the widespread use of these powerful molecules has led to the apparition of antibiotic-resistant bacterial strains. Silver NPs have broad spectrum activity against gram positive and gram negative bacteria (Agnihotri et al., 2014), biofilms (Guo et al., 2019), multidrug resistant bacteria (Lara et al., 2010a), fungi (Kim et al., 2009; Chudasama et al., 2011), and even some virus (Lara et al., 2010b). The antibacterial action of silver NPs is dependent on their size and shape (Choi and Hu, 2008).

Silver was initially used it its ionic form for biomedical applications, and, in particular, in surgical cloths and wound dressings (Uttayarat et al., 2012). Although silver ions can be toxic at high concentrations, great controversies exist regarding the toxicity of silver NPs, as this property depends on their size, shape, and surface functionalization. To our knowledge, no silver NP toxicity has been proven except the one resulting from the release of Ag^+^ ions. Therefore, silver NPs are actually being widely used as antibacterial agents (Reidy et al., 2013). Additionally, they can also be used for sensing applications (Annadhasan et al., 2014), as conductive elements in electronics (Cai et al., 2017b) and in environmental remediation applications as they possessed also catalytical properties allowing the degradation of several pollutants in water (Guerra et al., 2018).

#### Other Noble Metal NPs (Pd, Pt)

Platinum and palladium NPs are well-known in the catalysis field. Both metals are expensive which drives the industry interests toward the reduction of metal needed to obtain the same result. When used in their nanoparticulate form their catalytic performance increases due to the higher surface-to-volume ratio.

Platinum is mainly used in hydrogenation/dehydrogenation reactions, fuel cell applications, CO, and alcohol oxidations (Martínez-Prieto et al., 2017). On the other hand palladium NPs are used as catalyzers mainly in C-C coupling reactions (Suzuki–Miyaura, Sonogashira and Mizoroki-Heck reactions), reduction of nitroarenes, hydrogenation of alkenes and alkynes, and oxidation of primary alcohols (Saldan et al., 2015). Both metal NPs have been used as well for depollution applications (Iben Ayad et al., 2020), photothermal treatments (Samadi et al., 2018; Yang et al., 2019) and as antibacterial/antifungal agents (Dumas and Couvreur, 2015; Pedone et al., 2017).

### Quantum Dots

Quantum dots (QDs) are semiconductor NPs of small size (usually smaller than 10 nm). Because of their tunable fluorescent properties in a large light spectrum (from infrared to visible light), cadmium selenide (CdSe), cadmium telluride (CdTe), and cadmium sulfide (CdS) have been developed. In the biomedical field, QDs are used as biosensors, imaging probes and for diagnostics among others (Pandey and Bodas, 2020). QDs have been also used in photocatalysis applications. For example, the production of hydrogen using QD photocatalysis has been studied. This possibility is interesting for the use of hydrogen as an alternative combustible (Rao et al., 2019).

### Metal Oxide Nanoparticles

#### Iron Oxide NPs

Iron oxide NPs are of special interest because of their magnetic properties that differ from the bulk material. Similar to the LSPR effect, iron oxide particles present superparamagnetic behaviors in the nanoscale range (NPs of diameter below 20–30 nm). Because of their small size, these NPs act as single domain particles. They are magnetized in a uniform manner, with all the spins aligned in the same direction when a magnetic field is applied (Cardoso et al., 2018). Again, the magnetic properties of iron oxide NPs strongly depend on their size and shape, as well as their crystalline state (Demortière et al., 2011; Wu et al., 2016). The use of iron oxide NPs in the biomedical field is possible due to their biocompatibility and low toxicity. Because of their magnetic properties and their relaxation times, iron oxide NPs are good candidates for magnetic resonance imaging (MRI) contrast agents. Iron oxide NPs are a type T2 contrast agent resulting in a black contrast. These NPs are also used for protein, molecule or cell separation thanks to their magnetic properties (Cardoso et al., 2018; Cheng et al., 2019). Iron oxide NPs can be also used for hyperthermia treatments (Liang et al., 2019; Pires et al., 2019), drug delivery (Benyettou et al., 2019), tissue adhesion (Meddahi-Pellé et al., 2014) as magnetic stimulant (Santos et al., 2015), and as pollutant sorbents (Gutierrez et al., 2017).

#### Titanium Oxide NPs

Titanium oxide (TiO_2_) is a semiconductor metal used as a white pigment in paints, plastic, papers, cosmetics, food (E171), toothpaste among many others. This white appearance is due to its high refractive index (2.48–2.89) that results in the reflection of 96% of light. In addition, the high opacity of TiO_2_ NPs places it as the most used white pigment among industry. TiO_2_ is also the active component of sunscreens due to its capacity to absorb UV irradiation. Smaller NPs are preferred in this case as they result in colorless and fluid products (Lan et al., 2013). TiO_2_ NPs are also well-known because of their photocatalytic activity. Under ultraviolet irradiation, TiO_2_ electrons are excited and can be part of chemical reactions in the surface of the material. This property is mainly used for water hydrolysis to produce hydrogen, pollutant degradation (in air or water) resulting in auto-cleanable surfaces and bactericidal effect due to ROS production. Because of its biocompatibility, low cost, and high photocatalytic activity, TiO_2_ NPs are the most used photocatalyst (Haider et al., 2019), even if their reactivity in aqueous media is impacted by aggregation (Degabriel et al., 2018).

In the biomedical field, the photocatalytic activity of TiO_2_ NPs has been used for photodynamic treatment of infections, cancers and skin defects as psoriasis (Ahmad et al., 2015). However, this application is limited due to UV irradiation-induced damage in human tissues. To avoid direct use of UV radiation, NP surface modifications can be made, resulting in a shift in light absorption enabling the use of a different light source (Ni et al., 2017; Ziental et al., 2020). TiO_2_ NPs have been the subject of a vast literature regarding their toxicity, sometimes polemical (Horváth et al., 2018; Suzuki et al., 2020), and their interaction with proteins impacting cell behavior (Jayaram et al., 2017; Runa et al., 2017).

#### Other Metal Oxide NPs (Cu, Zn)

Other metal oxide NPs have been developed and are being used in industry. This group of NPs is used in many applications including antibacterial agents, gas sensors, catalysis, and electronics (Chavali and Nikolova, 2019). Zinc and copper oxide NPs are two of the most important ones aside from titanium oxide, and widely used in catalysis and as antibacterial agents (Stankic et al., 2016). Increasing research in the biomedical field raises the applications of metal oxide NPs as anticancer agents, anti-inflammatory and radiation protection among others (Augustine and Hasan, 2020).

### Hydroxyapatite, Bioactive Glass, and Silica NPs

Bioceramics, including hydroxyapatite (HAP), present low tensile strength and brittleness. This is an issue in bone tissue engineering applications, where biomechanical loads, including torsion, bending, compression, shear stress are frequently applied to the implanted scaffold. Further, their limited remodeling (Sun et al., 2009) and uncontrolled degradation *in vivo* can lead to changes in the extracellular environment, which can cause adverse effects including cell death (Ge et al., 2008). Polymer/hydroxyapatite nanocomposites offer a promising solution to these issues, by combining the tunable degradability of polymers like silk with the osteoconductivity of ceramic materials (Zhang et al., 2019). The composition of these composites can be tuned to present mechanical properties closer to that of native bone, while preserving biocompatibility and biosorption properties. The nanoscale features of HAP NPs are particularly advantageous because nanotopography modulates cell behaviors like adhesion, differentiation, and proliferation, by promoting greater protein interactions and therefore new bone formation (Van Der Sanden et al., 2010).

Bioactive glasses and glass-ceramics stimulate a beneficial response *in vivo* by bonding to the host bone tissue (Jones, 2013). Further, the controlled release of biologically active calcium and silica ions from these materials leads to the upregulation and activation of genes associated to osteoblastic differentiation, encouraging rapid bone regeneration (Jones, 2013). The controlled release of ions from the dissolution bioactive glasses can induce angiogenesis, opening up further possibilities for enhanced bone or soft tissue regeneration (Hench, 2009). Like with HAP, one of the primary limitations on the clinical use of bioactive glasses is the unpredictable behavior in complex physiological loading conditions. Incorporating them as biologically active phases into composite systems is a promising solution to overcome these challenges.

### Graphene Oxide

Graphene is an interesting nanomaterial owing to its high mechanical strength and outstanding electrical conductivity among others. However, its low solubility reduces its utilization possibilities. Instead, graphene oxide (GO) is easier to synthetize and has better solubility, while matching the mechanical strength of graphene. Further, the presence of chemical groups in GO allows its functionalization and interaction with surrounding molecules (Smith et al., 2019). Because of its electronic configuration, GO nanosheets are impenetrable by many different gases and so can be used as barriers for such molecules. The same molecule exclusion principle together with the high-water permeability of GO has been used to create water filters for wastewater treatment (Thebo et al., 2018). For the same reason, GO is also used as coating to avoid material corrosion (Smith et al., 2019). GO conformation is easily altered when exposed to humidity, light, or heat. This property allows the development of stimuli-responsive materials. For example, the presence of humidity results in GO swelling (Chen et al., 2017).

## Silk-Based Bionanocomposites

The promising potential of combining silk and NPs for the design of bionanocomposites with tailored properties and functions has been pursued. Nanocomposites can present new properties that are not achieved in a classical composite with the same materials. Bionanocomposites are nanocomposites containing a biological material such as collagen, cellulose, alginate or silk. They have been studied to develop replacement tissues, such as tendon (Yang et al., 2016), corneal stroma (Watanabe et al., 2020), bone (Raja and Yun, 2016) and dermis (Song et al., 2015).

However, these properties are only achieved if the NPs are homogeneously distributed within the resultant material. Therefore, when developing any type of nanocomposite, it is crucial to consider the NP surface chemistry, stabilization within the bulk material and homogeneous distribution. As previously explained, NP stabilization can be easily altered by changing the physicochemical conditions of their environment, such as ionic strength. Mixing NPs with other material results in a new environment so it is not surprising to observe NP aggregation and precipitation within the material. These considerations are particularly important for silk bionanocomposites as in this case silk gelation can easily occur as well due to NP addition to the solution.

**Figure 2:**
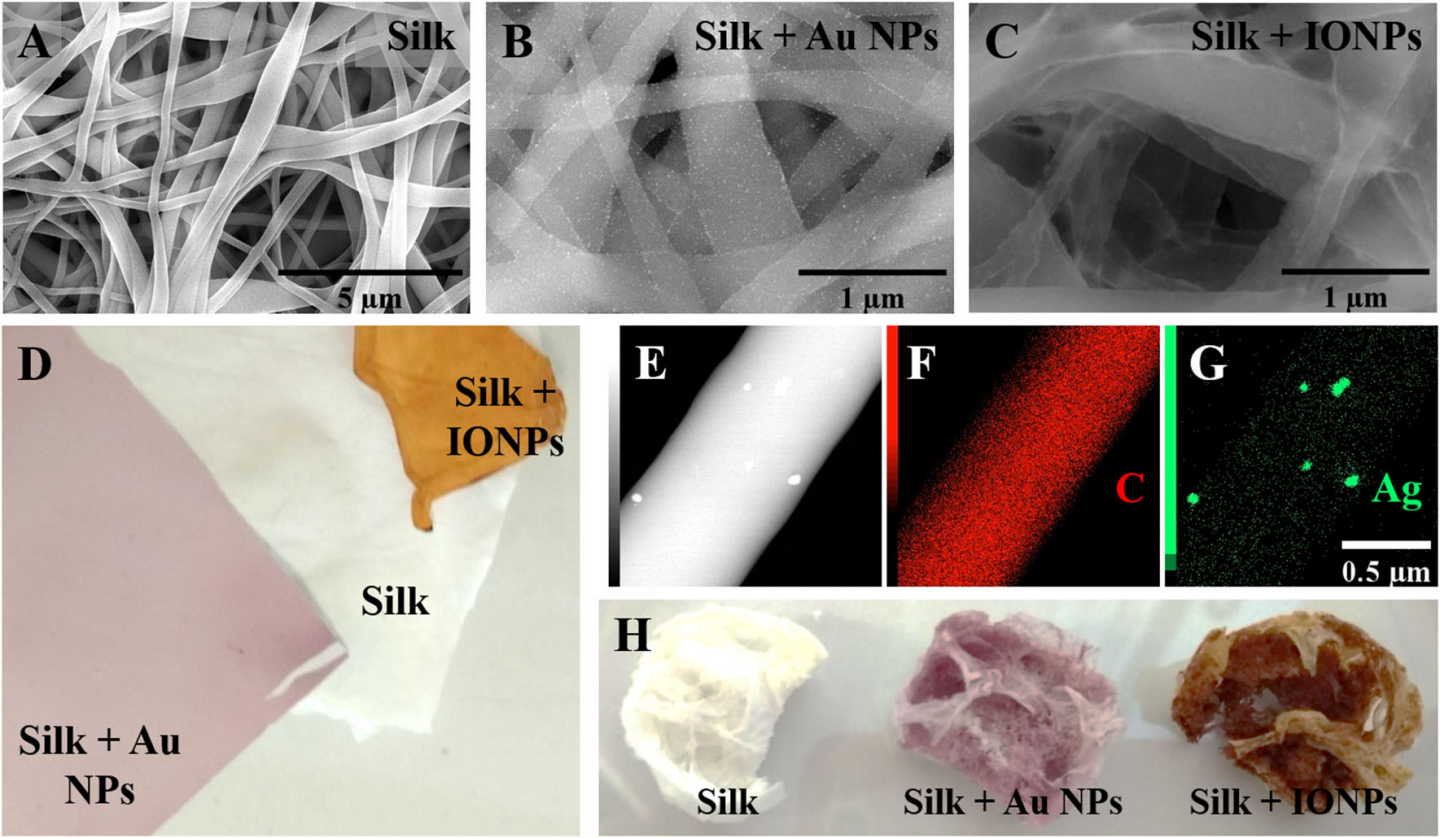
Examples of silk bionanocomposites. Scanning Electron Microscopy view of electrospun silk fibers **(A)** with gold nanoparticles (Au NPs) **(B)**, iron oxide nanoparticles (IONPs) **(C)** or silver nanoparticles (Ag NPs) [**(E–G)**, STEM-EDS with elemental mapping]. Macroscopic view of electrospun scaffolds **(D)** and sponges **(H)** with gold (Au NPs) and iron oxide nanoparticles (IONPs).

Silk bionanomaterials have been generated using at least three different methods. *In situ* synthesis was studied using many different reducing agents (Table 1), including the ability of silk to reduce metal ions (Yu et al., 2017; Zhou et al., 2020). Figures 2B–D show silk electrospun biocomposites obtained by *in situ* synthesis of gold (Au NPs) and iron oxide nanoparticles (IONPs). Although this approach reduces the number of steps needed to produce the bionanocomposite, the resultant NPs can be polydisperse in size and shape. Moreover, the surface chemistry of these NPs is unknown. These issues could result in unpredictable properties and toxicity, which are highly dependent on the characteristics of the NPs. Better control of NP characteristics can be achieved by synthesis upstream and subsequently incorporating them into silk materials. Figure 2 shows an example of silver NPs embedded in silk fibers (Figures 2E–G) and sponges (Figure 2H) obtained by using the latter procedure. However, in these cases it is important to stabilize NPs in solution by controlling their surface chemistry. Therefore, many studies have been conducted to further understand the stability of NPs in the solution (Hotze et al., 2010). Nevertheless, it is shown that, once included within the bulk material, the confinement of NPs enhances their stability by decreasing particle-particle interactions (Chandran et al., 2014). In addition, in many situations, the direct incorporation of NPs into regenerated silk solution induces silk gelation. Some studies have focused on feeding the desired NPs directly to silkworms, with incorporation in the silk cocoons, however, the efficiency of incorporation is low due to the NP biodistribution in the worm (Xu et al., 2019).

**Table 1:** Applications, NPs, functionalization method, and silk materials for silk-based bionanocomposites.

**Application**	**NPs type**	**Functionalization method**	**Silk material**	**References**
Antibacterial	Ag	*In situ*	Degummed fibers	Lu et al., 2014b; Meng et al., 2016
			Electrospun	Calamak et al., 2015
			Film	Yu et al., 2012, 2017; Raho et al., 2020
			Silk dispersion	Jia et al., 2017
			Sutures	Baygar et al., 2019
			Textile	Zhang et al., 2014; Shahid et al., 2017; Gao et al., 2019; Velmurugan et al., 2020
		*In situ* vs. upstream	Degummed fibers	Dhas et al., 2015
		Upstream	Electrospun	Uttayarat et al., 2012
			Fibers	Karthikeyan et al., 2011
			Textile	Gulrajani et al., 2008; Zhou and Tang, 2018a
	CuO	*In situ*	Yarn	Abbasi et al., 2016
	SeO	*In situ*	Electrospun	Chung et al., 2016
	ZnO	Upstream	Film	Patil et al., 2018
Antibacterial/catalysis/dyeing	Pt	*In situ*	Textile	Zou et al., 2018
Antibacterial/photocatalysis	Ag/AgCl		Porous films	Zhou et al., 2019
Antibacterial/tissue engineering	Ag Au HAP	*In situ*	Sponge	Ribeiro et al., 2017
	Ag			Hu et al., 2020
Antibacterial/wound healing	Ag	*In situ*	Gel	Patil et al., 2015
Antibacterial/UV protection	Au	*In situ*	Textile	Tang et al., 2014
	CeO_2_	Upstream	Degummed fibers	Lu et al., 2014a
Antibacterial tissue protection	Ag	Upstream	Textile	Zhou and Tang, 2018b
Antibiotic dose reduction	Au	*In situ*	Textile	Zhou et al., 2020
Catalysis	Au	*In situ*	Sponge	Das and Dhar, 2014
	Fe_3_O_4_		Hydrogel	Luo and Shao, 2017
	Pd		Degummed fibers	Ikawa et al., 2005
Depollution	CuO	*In situ*	Silk dispersion	Kim et al., 2017
	Fe_2_O_3_	Upstream	Cocoons	Liu et al., 2018
	TiO_2_	Upstream	Electrospun	Aziz et al., 2017
Hyperthermia	Au	Upstream	Hydrogel	Kojic et al., 2012
			Nanofibers	Wang J. et al., 2019
	Fe_3_O_4_		Hydrogel	Qian et al., 2020
Imaging	NaYF_4_@SiO_2_	Worm feeding	Cocoons	Deng et al., 2020
	C nanodots			Fan et al., 2019
	Au	*In situ*	Film	Yin et al., 2009a,b
			Silk dispersion	Ranjana et al., 2020
	C nanotubes/Au	Upstream	hydrogel	Zhang et al., 2020
	CdS	*In situ*	Fibers	Han et al., 2010
	CdTe	Upstream	Film	Sohail Haroone et al., 2018
	GO		Silk GO paper	Ma and Tsukruk, 2017
	Graphene		Film	Wang et al., 2017
	Ni nanodisc		Textile	Schmucker et al., 2014
Sensing SERS	Au	*In situ*	Silk textile	Liu et al., 2016
		Upstream	Film	Guo et al., 2015
Tissue engineering	Au	Upstream	Electrospun	Cohen-karni et al., 2012; Das et al., 2015; Sridhar et al., 2015; Akturk et al., 2016
	Bioactive glass		3D printed	Midha et al., 2018
			Sponge	Bidgoli et al., 2019
	CoFe_2_O_4_/Fe_3_O_4_		Electrospun	Brito-Pereira et al., 2018
	Cu bioactive glass		Hydrogel	Wu et al., 2019
	Fe_3_O_4_		Sponge	Aliramaji et al., 2017; Tanasa et al., 2020
	GO		Hydrogel	Wang et al., 2020
	HAP	*In situ*	3D printed	Huang et al., 2019
			Compacted powder	Zakharov et al., 2017
			Silk dispersion	Kong et al., 2004
		Upstream	3D printed	Sun et al., 2012
			Fibers	Heimbach et al., 2018
			Hydrogel	Ding et al., 2017
			Sponge	Kweon et al., 2011; Ye et al., 2017
	HAP TiO_2_	Upstream	Sponge	Kim et al., 2016
	Silica		Film	Mieszawska et al., 2010
Wearable electronics	Au	Upstream	Film	Tao et al., 2010

### Antibacterial Activity

In the textile world of silk, the incorporation of antibacterial NPs allows for improvement of luxury clothes and reduces odors. In the biomedical field, the presence of such NPs avoids, or at least delays, infections. Silver NPs are used for antibacterial applications and silk materials have been functionalized with silver NPs. UV irradiation was used to synthetize silver NPs directly in silk solutions (Jia et al., 2017), films (Zhou et al., 2019), sponges (Hu et al., 2020), fibers, textiles and electrospun mats (Calamak et al., 2015; Jia et al., 2017; Ribeiro et al., 2017; Zhou et al., 2019; Hu et al., 2020; Raho et al., 2020). *In situ* synthesis of NPs in silk textiles was not uniform over the fibers, likely due to a different silver ion adsorption depending on the silk chemical groups. Silver ion release was studied for electrospun mats and was dependent on β-sheet content. Electrospun materials with low β-sheet content resulted in cytotoxic effects in mammalian cells. Light was used as a reducing agent for the *in situ* synthesis of silver NPs in silk (Patil et al., 2015) and a gelling agent. The antibacterial activity of the silver NPs demonstrated. However, no tests were performed for the gel nanocomposite. The topical application of this gel in animal skin wound models resulted in faster wound closure in comparison with silk, silver NPs and Carbopol gels and Soframycin gel, a commercially available product (Patil et al., 2015). A difference was observed between silk and silver NPs/silk gels, suggesting a synergistic effect of both components in wound healing.

Silver NP-loaded silk hydrogels have been also used for bone regeneration (Ribeiro et al., 2017). Silver NPs were synthesized *in situ* using light as a reducing agent. Antibacterial activity was found for hydrogels containing more than 0.5% silver NPs. Cytocompatibility was assessed by seeding osteoblast cells on the hydrogels, while a silver NPs concentration-dependent decrease in cell viability was observed.

*In situ* NP synthesis in silk materials has also been developed using caffeic acid, flavonoids, vitamin C, citrate, *Rhizophora apiculata* leaf extracts, *Streptomyces* cell extracts or honeysuckle extracts as reducing agents (Dhas et al., 2015; Meng et al., 2016; Shahid et al., 2017; Zhou and Tang, 2018a,b; Baygar et al., 2019; Gao et al., 2019). The materials acquired antibacterial activities and showed no cytotoxic effects. Interestingly, the materials synthetized using caffeic acid had UV irradiation protection, suggesting applications in sun protective materials.

Silk sponges and films containing silver NPs have been developed (Yu et al., 2012; Das and Dhar, 2014). Silk alone was able to reduce Ag^+^ into Ag^0^ efficiently to form silver NPs. The reduction ability of SF, sericin and peptides has also been shown by others (Zhang et al., 2014; Yu et al., 2017; Zhou et al., 2020). All these *in situ* synthesis procedures resulted in NPs with uncontrolled sizes and shapes, parameters that are crucial to control in order to evaluate NP properties. In addition, no information about NP surface chemistry or the presence of remaining toxic silver ions was described. Altogether, materials obtained by this methodology may not possess the desired antibacterial properties and may also present undesired properties such as toxicity.

Mixing preformed NPs with silk has also been pursued (Gulrajani et al., 2008; Karthikeyan et al., 2011). A two-step functionalization of silk fabrics with silver NPs was reported (Gulrajani et al., 2008). The silk fabric was soaked in silver NPs solution and the effect of pH on NP uptake was assessed, finding greater adsorbtion in acidic media. Silk uptake of NPs was also temperature dependent, with improved adsorption at 40 vs. 80°C.

A two-step method was developed to functionalize silk fabrics with silver NPs (Zhou and Tang, 2018a). Interestingly the resulting materials were inhibited the growth of both *Escherichia coli* and *Staphylococcus aureus* even after 30 washes, suggesting a strong bond between the silk and silver NPs.

Other studies have focused on the antibacterial activity of gold (Ribeiro et al., 2017; Zhou et al., 2020), platinum (Zou et al., 2018), copper oxide (Abbasi et al., 2016), zinc oxide (Patil et al., 2018), cerium oxide (Lu et al., 2014a), selenium oxide NPs (Chung et al., 2016) in silk materials. The *in situ* synthesis of gold NPs in a HAP-containing silk hydrogel was carried out (Ribeiro et al., 2017). Once the hydrogel formed, gold NPs were synthesized by heating the solution to 60°C. Significant antibacterial activity was observed against *S. aureus* (Multidrug sensible and resistant strains), *E. coli* and *Pseudomonas aeruginosa*, but not against *Staphylococcus epidermidis*.

The *in situ* synthesis of gold NPs in silk fabrics was attained by heating to 85°C (Tang et al., 2014). The result was an antibacterial, UV irradiation blocking and thermal conducting material v for textile applications. However, as for the silver NPs, *in situ* synthesis does not allow tight control of NP size.

### Tissue Engineering

Different NPs containing silk materials have been developed for tissue engineering. Gold NPs can be incorporated into the silk electrospinning solution to obtain silk nanofibers with well-dispersed gold NPs by traditional (Cohen-karni et al., 2012) and wet (Akturk et al., 2016) electrospinning techniques. In both cases, the incorporation of gold NPs into electrospun silk materials resulted in improved mechanical properties. *In vitro* tests showed no cytotoxicity to the materials, and a good cell attachment to the scaffolds. In addition, cell attachment was enhanced by functionalizing the gold NPs with the integrin binding peptides RGD. The resultant materials were tested *in vivo* for wound closure (Akturk et al., 2016). No significant differences were found between silk with or without the gold NPs.

The incorporation of gold NPs into silk scaffolds has been also used to increase material conductivity. Electrical stimuli are crucial for nerve and cardiac tissues, thus, this modification impacts the regeneration process for these tissues. For example, electrospun silk containing gold NPs materials were rolled into conduits to replace sciatic nerves *in vivo* (Das et al., 2015). As a result, gold-containing silk materials outperformed pure silk materials in term of nerve regeneration. The presence of gold NPs in silk materials also supported better mesenchymal stem cell differentiation toward the cardiac lineage (Sridhar et al., 2015).

Most silk-based bionanocomposites developed for biomedical applications have focused on bone tissue regeneration. Because of their osteoconductive properties, silk materials containing HAP, bioactive ceramics and silica NPs have been studied. Nanocomposite scaffolds of silk and HAP are particularly interesting due to the ability of silk to “regulate” the mineralization of calcium phosphates, presumably through chemical interactions involving silk chemical groups (Kong et al., 2004; Zakharov et al., 2017). Silk/HAP scaffolds were manufactured using thermally induced phase separation (TIPS) (Wei and Ma, 2004) or lyophilization (Ye et al., 2017), to generate highly porous silk/nHAP composite scaffolds. Other materials used include silk sponges (Kweon et al., 2011), hydrogels (Ding et al., 2017), or 3D printed constructs (Huang et al., 2019).

Studies with silk/HAP materials for bone regeneration have shown promising results both *in vitro* and *in vivo* due to their biocompatibility, mechanical properties, and chemical composition similar to native bone. In a rabbit radial bone defect the formation of new bone tissue with a density similar to native bone was found using silk/chitosan/HAP biodegradable scaffolds obtained by lyophilization (Ye et al., 2017). A significantly higher rate of bone formation was also found in a rabbit parietal model after 8 weeks using silk sponges loaded with HAP (Kweon et al., 2011). Silk/HAP hydrogels have also been explored for the treatment of irregular bone defects by injection (Ding et al., 2017). *In vitro*, these hydrogels demonstrated good cytocompatibility and osteogenic differentiation capacity. *In vivo*, in a rat calvarial defect model, these hydrogels were able to support the formation of new bone tissue, suggesting promising applications for bone tissue engineering (Ding et al., 2017).

A bone replacement was developed using a silk hydrogel functionalized with hydroxyapatite NPs (nano-HAP) (Ribeiro et al., 2015). HAP is a calcium phosphate widely used in bone tissue engineering as its composition is close to the mineral phase of bone tissues. *In vitro* experiments showed that the nano-HAP-containing materials enhanced osteoblastic phenotype. The main challenge was to avoid nano-HAP aggregation during silk gelation. The material containing 15 wt% of nano-HAP showed a homogeneous dispersion in the silk hydrogel with no visible aggregation. The aggregation state of nano-HAP remains, however, the main constraint for the design of a silk-based materials with a higher nano-HAP contents.

Adequately matching the morphology of the implant and the surrounding bone is crucial for the proper integration of the implant with the surrounding bone. Recent work has focused on using or tuning the rheological properties of silk/HAP-based pastes for 3D printing applications. These approaches allow the formation of biomimetic and macroporous silk/HAP nanocomposite scaffolds. Using sodium alginate (SA) as a binder, 3D printed scaffolds with large, interconnected pores and relatively high compressive strength were generated and human bone marrow-derived mesenchymal stem cells (hMSCs) seeded on the scaffolds adhered, proliferated and differentiated toward an osteogenic lineage (Huang et al., 2019). Likewise, 3D printed scaffolds composed of a gradient of silk/hydroxyapatite (HA) were generated and supported the growth of hMSCs and human mammary microvascular endothelial cells (hMMECs) (Sun et al., 2012). These cells formed intricate networks of extracellular matrices within the 3D scaffolds with vascular-like structures following the scaffold morphology (Sun et al., 2012).

In addition to scaffold-based applications for bone regeneration, silk/HAP/polylactic acid composites were developed for the fabrication of high strength bioresorbable fixation devices for clinical applications in orthopedics (Heimbach et al., 2018). Bioactive glass NPs have been also used in bone tissue engineering using silk-based materials. For example, SF-bioactive glass composites were fabricated with controlled architecture and interconnected structure by combining indirect three-dimensional (3D) inkjet printing and freeze-drying methods. The silk/bioglass composite scaffolds possessed excellent mechanical properties and stability, and supported the attachment of hMSCs and possessed osteoinductive properties (Bidgoli et al., 2019). 3D printed silk-gelatin-bioactive glass hybrids were generated with the goal of developing patient-specific grafts for bone regeneration (Midha et al., 2018). Two different bioactive glass compositions were tested, with and without strontium, and the strontium-containing constructs induced osteogenic differentiation of MSCs. Further, ion release from bioactive glasses in the silk-gelatin ink triggered the activation of signaling pathways for *in vivo* bone formation (Midha et al., 2018). Bioactive glass nanoparticles can be functionalized with different ions before incorporation into silk matrices. For example, injectable composite hydrogels of chitosan, silk and glycerophosphate were developed and were highly porous and could be used to administer silica, calcium and copper ions in a sustained and controlled manner (Wu et al., 2019). Further, these materials were suitable for *in situ* injection and underwent rapid gelation under physiological conditions of temperature and pH. *In vitro*, these hydrogels supported the growth of MC3T3-E1 and human umbilical vein endothelial cells, and induced them toward osteogenesis and angiogenesis, respectively. *In vivo*, the copper-functionalized hydrogels restored critical-size rat calvarial bone defects with the newly formed bone tissue vascularized with mineralized collagen deposition in 8 weeks (Wu et al., 2019).

In addition, incorporating silica NPs in silk matrices to confer osteoinductive properties, silica-functionalized silk films were prepared where hMSCs adhered, proliferated, and differentiated toward an osteogenic lineage (Mieszawska et al., 2010). The presence of silica led to upregulation of osteogenic gene expression, including bone sialoprotein and collagen type 1. Collagen fibers and apatite nodules were observed, indicating the formation of new mineralized bone tissue (Mieszawska et al., 2010).

Other NPs have also been incorporated into silk materials for bone tissue engineering. For example TiO_2_ NPs or GO have been used to increase the mechanical resistance of silk sponges and hydrogels (Kim et al., 2016; Wang et al., 2020). Magnetic NPs have interesting properties that can be used to apply a magnetic stimuli to cells and tissues (Aliramaji et al., 2017; Brito-Pereira et al., 2018; Tanasa et al., 2020). Recently a silk chitosan magnetite bionanocomposite scaffold was prepared by freeze casting (Aliramaji et al., 2017). They addition of magnetite NPs to the silk/chitosan scaffold did not change the porosity and no magnetite release was detected in a PBS solution after 48 h, to support the stability of NPs in the scaffolds. The combination of magnetite NPs within the scaffolds, together with the static magnetic field applied resulted in no osteosarcoma cell cytotoxicity and increased cell attachment.

Overall, the results described above demonstrate the potential of NP incorporation in silk as a tool for material functionalization; improving mechanical properties, and also to serve as biochemical cues for the surrounding cells toward bone tissue engineering. As our understanding of mechanisms and signaling pathways progresses, the regeneration of complex tissues may benefit from combinations of NPs in silk scaffolding, including spatial patterning, to induce improved and perhaps patterned regeneration outcomes.

### Hyperthermia

Hyperthermia as a therapy for the treatment of cancer uses various external energies, such as microwave, ultrasound, infrared radiations, to locally increase the body temperature and therefore destroy tumor cells. The heaters can be plasmonic or superparamagnetic NPs (Chicheł et al., 2007; Kolosnjaj-Tabi et al., 2017). Among hyperthermia modalities, phototheraphy is an interesting non-invasive therapy, in which light is used to induce local cell death. Due to the intrinsic light absorption of biological tissues, photothermal therapies can be only achieved in the near infrared region (NIR). State-of-the-art photothermal agents, namely gold nanorods and nanostars, are not efficient in the NRI II transparency window.

Silk nanofibers containing gold NPs were developed (Wang J. et al., 2019). The specific assembly of NPs results in the broader absorption of light in the NIR and a red shift of the maximum. As a result, gold NP-containing silk nanofibers reached higher temperatures than gold NPs alone under the same conditions. Therefore, the ability to pattern how and where NPs adhere to silk materials result in increased efficiency (Plan Sangnier et al., 2020).

Differently, injectable silk hydrogels containing gold NPs have also been developed for photothermal treatments of infections (Kojic et al., 2012). Silk hydrogels were obtained by vortexing silk solutions in which gold NPs were incorporated. The hydrogels were injected in the infected site and heat was produced by laser exposition. Interestingly heat was produced locally and was able to reduce bacterial populations, thus, reducing infection. The silk hydrogel assured spatial localization of the gold NPs at the injection site. Light penetration limits in biological structures can be overcome by using magnetic fields for hyperthermia treatments. In this case magnetic nanoparticles are placed in the treatment zone and heat is produced in an external magnetic field (Sohail et al., 2017). Injectable silk hydrogels have been formulated for intratumoral injection using this strategy (Qian et al., 2020). The application of a magnetic field successfully enabled deep tumor ablation while no damage was observed in the surrounding healthy tissue. Furthermore, the magnetic field can be used to direct the material to the target spot, reducing systemic distribution of the magnetic NPs.

### Imaging

The possibility to follow silk implants by non-invasive imaging can be achieved by introducing fluorescent or contrast agents into the material. As an example, the introduction of iron oxide NPs into silk materials allows the use of MRI for visualization *in vivo* (Qian et al., 2020). NIR emitting NaYF_4_@SiO_2_ NPs were synthetized and silk cocoons were functionalized by directly feeding silk worms with the NPs (Deng et al., 2020). The resultant silk materials were visible by NIR II imaging once implanted into mice. Similarly, silkworms were fed with fluorescent carbon nanodots resulting in fluorescent silk fibers (Fan et al., 2019). Although the materials showed no cytotoxicity, the fluorescent capacity was not evaluated *in vivo*. These results are encouraging for the *in vivo* monitoring of silk implants.

### Electronics and Sensing

The transparency, flexibility, biodegradability and biocompatibility properties of silk materials have been used to develop wearable electrodes and sensors. In many cases, NPs are incorporated to support increased detection sensitivity. As in hyperthermia, gold NPs-containing silk materials have been coupled to thermoelectric chips. By doing so and incorporating the chip into an implantable device, light can be used as an energy source (Tao et al., 2010).

Silk sensors have been developed to detect ammonia (Ranjana et al., 2020) and immunoglobulin G (Sohail Haroone et al., 2018). For example, the detection of ammonia was achieved with *in situ*-synthetized gold NPs in a silk dispersion with UV-B light as reducing agent (Ranjana et al., 2020). Interestingly, the UV absorption of gold NPs decreased as the ammonia concentration increased. However, no control of the size and shape of the NPs was observed, and absorbance is dependent on these features. Silk materials have also been used to protect enzyme-mediated biosensors for the detection of hydrogen peroxide (Yin et al., 2009a), as well as methyl paraoxon, carbofuran and phoxim (Yin et al., 2009b).

Gold NPs-embedded silk films have been developed to enhance Surface-Enhanced Raman Spectroscopy (SERS) signals (Guo et al., 2015). The signal enhancement factor from the silk/gold NP films was around 150-fold. However, t light absorption by the gold NPs was influenced by the presence of the silk and red shifted by 20 nm. This result indicated a silk-induced change in the optical properties of the gold NPs, possibly due to the different refractive indexes of silk and the aqueous solution. The results suggest a capability of these films to be used as biosensors. Similar findings based on *in situ*-synthetized gold NPs in silk fabrics have been reported (Liu et al., 2016).

Silk hydrogels containing carbon nanotubes have been developed to respond to mechanical stimuli (Zhang et al., 2020). The composites were able to sense pressure variations, bending and compression forces. These abilities are interesting for medical applications such as to monitor arterial pressure and intracranial pressure. Gold NPs were integrated into the system, resulting in a laser-mediated degradation due to heat production. Altogether the silk hydrogels were able to trigger a laser exposition-mediated degradation when detecting epileptic episodes (mechanical stimuli). The incorporation of drugs into the hydrogel allows for controlled therapy.

Microcircuits were printed on a silk/GO-based paper (Ma and Tsukruk, 2017). The possibility to have a well-designed circuit structure allows the fine-tuning of electrode responses to external stimuli such as changes in relative humidity or proximity sensing. Silk materials were also used to prevent fluorescence quenching due to QD aggregation (Han et al., 2010). The immobilization of CdTe QDs increased fluorescence lifetime and IgG sensing capability. Silk fibers have also been used to direct the arrangement and *in situ* synthesis of CdS QDs for photoluminescence applications (Han et al., 2010). Surprisingly, physical and chemical nanotags for anti-counterfeiting applications have also been developed where nickel nanodisc structures with or without chromophores were embedded into silk textiles by electrospinning (Schmucker et al., 2014). The unlimited combinations of structure and chromophores enabled the generation of multiple tags identifying different products.

### Catalysis

Currently, nanocatalysts are used for industrial applications due to the increased surface-to-volume ratio, allowing the same catalytic activity as in bulk materials but requiring less catalyst. However, because of their nanometric dimensions, their collection for reutilization is particularly difficult. Therefore, there is an increasing interest in immobilizing nanomaterials into supports, and silk has been used for its biodegradation and biocompatible features. Catalysis activities of platinum (Zou et al., 2018), gold (Das and Dhar, 2014), palladium (Ikawa et al., 2005), and iron oxide NPs (Luo and Shao, 2017) have been studied in silk materials.

Silk sponges and films containing gold NPs were developed for catalysis using the same preparation methodology used for silk materials containing silver NPs (Das and Dhar, 2014). The reduction of 4-nitrophenol catalyzed by gold NPs containing silk materials was demonstrated. Iron oxide NPs were synthetized *in situ* in silk materials using silk hydrogels (Luo and Shao, 2017). A co-precipitation methodology was used to prepare the embedded NPs-embedded by dipping the hydrogel into a solution containing FeCl_2_ and FeCl_3_, and adding ammonium hydroxide to trigger NP synthesis. The magnetic and catalytic activities of magnetite were preserved in the silk materials. Such materials could be used in environmental chemistry applications and be easily separated by their magnetic properties; however, their use in biological applications is compromised by the presence of ammonium hydroxide. Interestingly, the immobilization of palladium nanoparticles into silk materials conserved its catalytic activity and also enhanced the chemoselectivity of the hydrogenation catalyzed reaction (Ikawa et al., 2005).

### Pollution Control

In addition to mechanical strength, biocompatibility and biodegradability of silk, this material is also a good absorbent for aromatic dyes. Therefore, the combination of absorbent properties, dependent on pH and dye concentration, with catalytical activity of several NPs has been explored.

The combination of silk electrospun nanofilters with TiO_2_ NPs for anionic dye removal was studied (Aziz et al., 2017). Interestingly, the absorption capacities of the materials increased as the NP content increased, and better absorption was achieved at acidic pH. Similarly, silk iron oxide NPs materials were developed for anionic dye removal (Liu et al., 2018). The photocatalytic activity of CuO_2_ NPs embedded in silk for dye removal has also been explored (Kim et al., 2017). These results provide insights into wastewater treatments with biodegradable materials like silk, functionalized with suitable NPs. The combination of SF with hydroxyapatite was efficient for water filtration and purification (Ling et al., 2017). The material was able to remove efficiently dyes and heavy metal ions from solution, a result not achieved with conventional nanofiltration membranes. Silk can also be mixed with silica to form insulating and fire retardant materials (Maleki et al., 2018). These materials can be obtained by a one-step acid catalyzed sol-gel reaction. The resulting silk/silica aerogel showed low density (0.11–0.19 g/cm^3^), high surface area (311–798 m^2^/g), flexibility in compression, and fire retardancy.

## Perspectives and Future Outlooks

The great versatility of silk bionanocomposites, due to the possibility to design different material types and embed a variety of inorganic nanomaterials, is at the origin of their outstanding tailored properties and fucntion. Therefore, a better match within the material and the specific requirements for a given application is possible. In the biomedical field, the development of personalized silk bionanocomposite materials becomes more and more feasible. However, many issues are still to be addressed, particularly the long-term storage and batch-to-batch differences. The fate of the materials for long-term applications deserve, indeed, further investigations, to evaluate the evolution of mechanical properties due to aging, the stability of NPs and their possible release, etc. This requires in-depth physicochemical characterizations, on the one hand, and a great number of clinical trials, on the other hand. For the emerging applications, such as pollution control, silk bionanocomposites are promising as they enable the combination of pollutant adsorption and catalysis procedures.

## Conclusions

Our walkthrough of the state-of-the-art supports the versatility of silk in a range of materials and applications for these materials. Silk can be shaped into many different material formats in a tunable manner. The versatility allows for easy adaptation to each application, as well as the possibility to combine silk with different NPs for new properties.

However, silk-based bionanocomposites still have some drawbacks to overcome. Two main strategies for silk bionanocomposite preparation are used: *in situ* synthesis and the addition of previously synthetized NPs (upstream) to silk materials. *In situ* synthesis methods are promising by reducing the reaction steps to obtain the final functionalized materials, such as the successful for HAP/silk bionanocomposites obtained by controlled calcium phosphate mineralization. However, silk-based bionanocomposites often fail to control NP size and shape efficiently, which determine the function of the materials. The synthesis of NPs prior to addition to silk materials raises other questions inherent to NP synthesis, control of size, surface functionality and dispersion. Although advances have been made in NP synthesis methodologies, the synthesis of NPs needs to: (i) have control over size and shape; (ii) stabilized NPs in the surrounding media and avoiding NP aggregation or precipitation; (iii) control of surface chemistry of NPs. Finally, the addition of synthesized NPs can induce silk gelation. Overall, the bionanocomposites should have NPs of controlled size, shape and surface chemistry, and these NPs should be homogeneously distributed within the silk matrix.

Silk bionanocomposites have been largely developed for antibacterial and tissue engineering applications because of their biocompatibility and biodegradation properties. Antibacterial properties allow interesting textile conservation and wound protection from infections. Moreover, the versatility given by different silk materials and the combinations with nanocomponents are well-adapted to many applications in tissue engineering. Emerging applications of silk bionancomposites have also been described in many other fields such as catalysis, electronics, imaging and sensing devices.

In the future, different combinations of silk/NPs materials may be developed for additional applications. In addition, with the increasing concern on climate change and plastic pollution, biodegradable materials based on silk should remain in the spotlight to provide new materials for new functions in medicine, for the environment, and for a range of additional needs. Improved control of NP dimensions and homogeneity will further drive this innovation in next generation silk-based materials.

## Author Contributions

All authors listed have made a substantial, direct and intellectual contribution to the work, and approved it for publication.

## Conflict of Interest

The authors declare that the research was conducted in the absence of any commercial or financial relationships that could be construed as a potential conflict of interest.

## References

[B1] Abbasi A. R. Noori N. Azadbakht A. Bafarani M. (2016). Dense coating of surface mounted Cu_2_O nanoparticles upon silk fibers under ultrasound irradiation with antibacterial activity. J. Iran. Chem. Soc. 13, 1273–1281. 10.1007/s13738-016-0841-y

[B2] Agnihotri S. Mukherji S. Mukherji S. (2014). Size-controlled silver nanoparticles synthesized over the range 5-100 nm using the same protocol and their antibacterial efficacy. RSC Adv. 4, 3974–3983. 10.1039/C3RA44507K

[B3] Ahmad R. Mohsin M. Ahmad T. Sardar M. (2015). Alpha amylase assisted synthesis of TiO_2_ nanoparticles: structural characterization and application as antibacterial agents. J. Hazard. Mater. 283, 171–177. 10.1016/j.jhazmat.2014.08.073 25270329

[B4] Akturk O. Kismet K. Yasti A. C. Kuru S. Duymus M. E. Kaya F. (2016). Wet electrospun silk fibroin/gold nanoparticle 3D matrices for wound healing applications. RSC Adv. 6, 13234–13250. 10.1039/C5RA24225H

[B5] Alessandrino A. Chiarini A. Biagiotti M. Dal Prà I. Bassani G. A. Vincoli V. (2019). Three-layered silk fibroin tubular scaffold for the repair and regeneration of small caliber blood vessels: from design to *in vivo* pilot tests. Front. Bioeng. Biotechnol. 7, 1–17. 10.3389/fbioe.2019.00356 31850325 PMC6895545

[B6] Aliramaji S. Zamanian A. Mozafari M. (2017). Super-paramagnetic responsive silk fibroin/chitosan/magnetite scaffolds with tunable pore structures for bone tissue engineering applications. Mater. Sci. Eng. C 70, 736–744. 10.1016/j.msec.2016.09.039 27770949

[B7] Alshammari A. S. (2019). Heterogeneous gold catalysis: from discovery to applications. Catalysts 9:402 10.3390/catal9050402

[B8] Annadhasan M. Muthukumarasamyvel T. Sankar Babu V. R. Rajendiran N. (2014). Green synthesized silver and gold nanoparticles for colorimetric detection of Hg^2+^, Pb^2+^, and Mn^2+^ in aqueous medium. ACS Sustain. Chem. Eng. 2, 887–896. 10.1021/sc400500z

[B9] Augustine R. Hasan A. (2020). Emerging applications of biocompatible phytosynthesized metal/metal oxide nanoparticles in healthcare. J. Drug Deliv. Sci. Technol. 56:101516 10.1016/j.jddst.2020.101516

[B10] Aziz S. Sabzi M. Fattahi A. Arkan E. (2017). Electrospun silk fibroin/PAN double-layer nanofibrous membranes containing polyaniline/TiO_2_ nanoparticles for anionic dye removal. J. Polym. Res. 24, 0–6. 10.1007/s10965-017-1298-0

[B11] Balfourier A. Luciani N. Wang G. Lelong G. Ersen O. Khelfa A. (2020). Unexpected intracellular biodegradation and recrystallization of gold nanoparticles. Proc. Natl. Acad. Sci. U.S.A. 117, 103–113. 10.1073/pnas.1911734116 31852822 PMC6955300

[B12] Bandyopadhyay A. Chowdhury S. K. Dey S. Moses J. C. Mandal B. B. (2019). Silk: a promising biomaterial opening new vistas towards affordable healthcare solutions. J. Indian Inst. Sci. 99, 445–487. 10.1007/s41745-019-00114-y

[B13] Bang S. Ko Y. G. Kim W. Il Cho D. Park W. H. Kwon O. H. (2017). Preventing postoperative tissue adhesion using injectable carboxymethyl cellulose-pullulan hydrogels. Int. J. Biol. Macromol. 105, 886–893. 10.1016/j.ijbiomac.2017.07.103 28729217

[B14] Baygar T. Sarac N. Ugur A. Karaca I. R. (2019). Antimicrobial characteristics and biocompatibility of the surgical sutures coated with biosynthesized silver nanoparticles. Bioorg. Chem. 86, 254–258. 10.1016/j.bioorg.2018.12.034 30716622

[B15] Begum R. Najeeb J. Sattar A. Naseem K. Irfan A. Al-Sehemi A. G. (2019). Chemical reduction of methylene blue in the presence of nanocatalysts: a critical review. Rev. Chem. Eng. 0, 749–770. 10.1515/revce-2018-0047

[B16] Belanger K. Schlatter G. Hébraud A. Marin F. Testelin S. Dakpé S. (2018). A multi-layered nerve guidance conduit design adapted to facilitate surgical implantation. Heal. Sci. Reports 1:e86 10.1002/hsr2.86 PMC6295612 30623049

[B17] Bellas E. Lo T. J. Fournier E. P. Brown J. E. Abbott R. D. Gil E. S. (2015). Injectable silk foams for soft tissue regeneration. Adv. Healthc. Mater. 4, 452–459. 10.1002/adhm.201400506 25323438 PMC4399489

[B18] Benyettou F. Prakasam T. Ramdas Nair A. Witzel I. I. Alhashimi M. Skorjanc T. (2019). Potent and selective *in vitro* and *in vivo* antiproliferative effects of metal-organic trefoil knots. Chem. Sci. 10, 5884–5892. 10.1039/C9SC01218D 31360392 PMC6582759

[B19] Bhattacharjee P. Kundu B. Naskar D. Kim H. W. Maiti T. K. Bhattacharya D. (2017). Silk scaffolds in bone tissue engineering: an overview. Acta Biomater. 63, 1–17. 10.1016/j.actbio.2017.09.027 28941652

[B20] Bi B. Liu H. Kang W. Zhuo R. Jiang X. (2019). An injectable enzymatically crosslinked tyramine-modified carboxymethyl chitin hydrogel for biomedical applications. Colloids Surfaces B Biointerfaces 175, 614–624. 10.1016/j.colsurfb.2018.12.029 30583217

[B21] Bidgoli M. R. Alemzadeh I. Tamjid E. Khafaji M. Vossoughi M. (2019). Fabrication of hierarchically porous silk fibroin-bioactive glass composite scaffold via indirect 3D printing: effect of particle size on physico-mechanical properties and *in vitro* cellular behavior. Mater. Sci. Eng. C 103:109688 10.1016/j.msec.2019.04.067 31349405

[B22] Boopathy A. V. Mandal A. Kulp D. W. Menis S. Bennett N. R. Watkins H. C. (2019). Enhancing humoral immunity via sustained-release implantable microneedle patch vaccination. Proc. Natl. Acad. Sci. U.S.A. 116, 16473–16478. 10.1073/pnas.1902179116 31358641 PMC6697788

[B23] Brito-Pereira R. Correia D. M. Ribeiro C. Francesko A. Etxebarria I. Pérez-Álvarez L. (2018). Silk fibroin-magnetic hybrid composite electrospun fibers for tissue engineering applications. Compos. Part B Eng. 141, 70–75. 10.1016/j.compositesb.2017.12.046

[B24] Brown J. E. Moreau J. E. Berman A. M. McSherry H. J. Coburn J. M. Schmidt D. F. (2017). Shape memory silk protein sponges for minimally invasive tissue regeneration. Adv. Healthc. Mater. 6:e201600762 10.1002/adhm.201600762 PMC5266640 27863133

[B25] Cai Y. Guo J. Chen C. Yao C. Chung S. M. Yao J. (2017a). Silk fibroin membrane used for guided bone tissue regeneration. Mater. Sci. Eng. C 70, 148–154. 10.1016/j.msec.2016.08.070 27770874

[B26] Cai Y. Piao X. Gao W. Zhang Z. Nie E. Sun Z. (2017b). Large-scale and facile synthesis of silver nanoparticles: via a microwave method for a conductive pen. RSC Adv. 7, 34041–34048. 10.1039/C7RA05125E

[B27] Calamak S. Aksoy E. A. Ertas N. Erdogdu C. Sagiroglu M. Ulubayram K. (2015). Ag/silk fibroin nanofibers: effect of fibroin morphology on Ag^+^ release and antibacterial activity. Eur. Polym. J. 67, 99–112. 10.1016/j.eurpolymj.2015.03.068

[B28] Cardoso V. F. Francesko A. Ribeiro C. Bañobre-López M. Martins P. Lanceros-Mendez S. (2018). Advances in magnetic nanoparticles for biomedical applications. Adv. Healthc. Mater. 7, 1–35. 10.1002/adhm.201700845 29280314

[B29] Chambre L. Parker R. N. Allardyce B. J. Valente F. Rajkhowa R. Dilley R. J. (2020). Tunable biodegradable silk-based memory foams with controlled release of antibiotics. ACS Appl. Bio Mater. 3, 2466–2472. 10.1021/acsabm.0c00186 35025296

[B30] Chandran S. Begam N. Padmanabhan V. Basu J. K. (2014). Confinement enhances dispersion in nanoparticle-polymer blend films. Nat. Commun. 5, 1–9. 10.1038/ncomms4697 24805269

[B31] Chavali M. S. Nikolova M. P. (2019). Metal oxide nanoparticles and their applications in nanotechnology. SN Appl. Sci. 1, 1–30. 10.1007/s42452-019-0592-3

[B32] Chen L. Weng M. Zhou P. Zhang L. Huang Z. Zhang W. (2017). Multi-responsive actuators based on a graphene oxide composite: intelligent robot and bioinspired applications. Nanoscale 9, 9825–9833. 10.1039/C7NR01913K 28585961

[B33] Cheng F. Zhu C. He W. Zhao J. Qu J. (2019). PSBMA-conjugated magnetic nanoparticles for selective IgG separation. Langmuir 35, 1111–1118. 10.1021/acs.langmuir.8b00878 29792033

[B34] Chicheł A. Skowronek J. Kubaszewska M. Kanikowski M. (2007). Hyperthermia - description of a method and a review of clinical applications. Reports Pract. Oncol. Radiother. 12, 267–275. 10.1016/S1507-1367(10)60065-X

[B35] Choi O. Hu Z. (2008). Size dependent and reactive oxygen species related nanosilver toxicity to nitrifying bacteria. Environ. Sci. Technol. 42, 4583–4588. 10.1021/es703238h 18605590

[B36] Chudasama B. Vala A. K. Andhariya N. Upadhyay R. V. Mehta R. V. (2011). Antifungal activity of multifunctional Fe_3_O_4_-Ag nanocolloids. J. Magn. Magn. Mater. 323, 1233–1237. 10.1016/j.jmmm.2010.11.012

[B37] Chung S. Ercan B. Roy A. K. Webster T. J. (2016). Addition of selenium nanoparticles to electrospun silk scaffold improves the mammalian cell activity while reducing bacterial growth. Front. Physiol. 7, 1–6. 10.3389/fphys.2016.00297 27471473 PMC4943957

[B38] Cohen-karni T. Jeong K. J. Tsui J. H. Reznor G. Mustata M. Wanunu M. (2012). Nanocomposite gold-silk nano fibers. Nano Lett. 12, 5403–5406. 10.1021/nl302810c 22928701 PMC3468663

[B39] Cui Y. Zhao Y. Tian Y. Zhang W. Lü X. Jiang X. (2012). The molecular mechanism of action of bactericidal gold nanoparticles on *Escherichia coli*. Biomaterials 33, 2327–2333. 10.1016/j.biomaterials.2011.11.057 22182745

[B40] Das S. Dhar B. B. (2014). Green synthesis of noble metal nanoparticles using cysteine-modified silk fibroin: catalysis and antibacterial activity. RSC Adv. 4, 46285–46292. 10.1039/C4RA06179A

[B41] Das S. Sharma M. Saharia D. Sarma K. K. Sarma M. G. Borthakur B. B. (2015). *In vivo* studies of silk based gold nano-composite conduits for functional peripheral nerve regeneration. Biomaterials 62, 66–75. 10.1016/j.biomaterials.2015.04.047 26026910

[B42] De Crozals G. Bonnet R. Farre C. Chaix C. (2016). Nanoparticles with multiple properties for biomedical applications: a strategic guide. Nano Today 11, 435–463. 10.1016/j.nantod.2016.07.002

[B43] Degabriel T. Colaço E. Domingos R. F. El Kirat K. Brouri D. Casale S. (2018). Factors impacting the aggregation/agglomeration and photocatalytic activity of highly crystalline spheroid- and rod-shaped TiO_2_ nanoparticles in aqueous solutions. Phys. Chem. Chem. Phys. 20, 12898–12907. 10.1039/C7CP08054A 29700516

[B44] Demortière A. Panissod P. Pichon B. P. Pourroy G. Guillon D. Donnio B. (2011). Size-dependent properties of magnetic iron oxide nanocrystals. Nanoscale 3, 225–232. 10.1039/C0NR00521E 21060937

[B45] Demuth P. C. Min Y. Irvine D. J. Hammond P. T. (2014). Implantable silk composite microneedles for programmable vaccine release kinetics and enhanced immunogenicity in transcutaneous immunization. Adv. Healthc. Mater. 3, 47–58. 10.1002/adhm.201300139 23847143 PMC3950936

[B46] Deng Z. Huang J. Xue Z. Jiang M. Li Y. Zeng S. (2020). A general strategy for designing NIR-II emissive silk for the *in vivo* monitoring of an implanted stent model beyond 1500 nm. J. Mater. Chem. B 8, 4587–4592. 10.1039/C9TB02685A 32348399

[B47] Dhas S. P. Anbarasan S. Mukherjee A. Chandrasekaran N. (2015). Biobased silver nanocolloid coating on silk fibers for prevention of post-surgical wound infections. Int. J. Nanomedicine 10, 159–170. 10.2147/IJN.S82211 26491317 PMC4599606

[B48] Ding Z. Han H. Fan Z. Lu H. Sang Y. Yao Y. (2017). Nanoscale silk-hydroxyapatite hydrogels for injectable bone biomaterials. ACS Appl. Mater. Interfaces 9, 16913–16921. 10.1021/acsami.7b03932 28471165

[B49] Dumas A. Couvreur P. (2015). Palladium: a future key player in the nanomedical field? Chem. Sci. 6, 2153–2157. 10.1039/C5SC00070J 28694948 PMC5485570

[B50] Fan S. Zheng X. Zhan Q. Zhang H. Shao H. Wang J. (2019). Super-strong and intrinsically fluorescent silkworm silk from carbon nanodots feeding. Nano-Micro Lett. 11, 1–11. 10.1007/s40820-019-0303-z PMC7770652 34138020

[B51] Fernández-García L. Marí-Buyé N. Barios J. A. Madurga R. Elices M Pérez-Rigueiro J. (2016). Safety and tolerability of silk fibroin hydrogels implanted into the mouse brain. Acta Biomater. 45, 262–275. 10.1016/j.actbio.2016.09.003 27592819

[B52] Frauchiger D. A. Tekari A. Wöltje M. Fortunato G. Benneker L. M. Gantenbein B. (2017). A review of the application of reinforced hydrogels and silk as biomaterials for intervertebral disc repair. Eur. Cells Mater. 34, 271–290. 10.22203/eCM.v034a17 29064532

[B53] Gao A. Chen H. Hou A. Xie K. (2019). Efficient antimicrobial silk composites using synergistic effects of violacein and silver nanoparticles. Mater. Sci. Eng. C 103:109821 10.1016/j.msec.2019.109821 31349531

[B54] Gao X. Gou J. Zhang L. Duan S. Li C. (2018). A silk fibroin based green nano-filter for air filtration. RSC Adv. 8, 8181–8189. 10.1039/C7RA12879G PMC9078515 35542020

[B55] Ge Z. Jin Z. Cao T. (2008). Manufacture of degradable polymeric scaffolds for bone regeneration. Biomed. Mater. 3:022001 10.1088/1748-6041/3/2/022001 18523339

[B56] Gil E. S. Mandal B. B. Park S. H. Marchant J. K. Omenetto F. G. and Kaplan D. L. (2010). Helicoidal multi-lamellar features of RGD-functionalized silk biomaterials for corneal tissue engineering. Biomaterials 31, 8953–8963. 10.1016/j.biomaterials.2010.08.017 20801503 PMC2949540

[B57] Gil E. S. Panilaitis B. Bellas E. Kaplan D. L. (2013). Functionalized silk biomaterials for wound healing. Adv. Healthc. Mater. 2, 206–217. 10.1002/adhm.201200192 23184644 PMC3735446

[B58] Guerra F. D. Attia M. F. Whitehead D. C. Alexis F. (2018). Nanotechnology for environmental remediation: materials and applications. Molecules 23, 1–23. 10.3390/molecules23071760 PMC6100491 30021974

[B59] Gulrajani M. L. Gupta D. Periyasamy S. Muthu S. G. (2008). Preparation and application of silver nanoparticles on silk for imparting antimicrobial properties. J. Appl. Polym. Sci. 108, 614–623. 10.1002/app.27584

[B60] Guo C. Hall G. N. Addison J. B. Yarger J. L. (2015). Gold nanoparticle-doped silk film as biocompatible SERS substrate. RSC Adv. 5, 1937–1942. 10.1039/C4RA11051J

[B61] Guo C. Li C. Vu H. V. Hanna P. Lechtig A. Qiu Y. (2020). Thermoplastic moulding of regenerated silk. Nat. Mater. 19, 102–108. 10.1038/s41563-019-0560-8 31844276 PMC6986341

[B62] Guo J. Qin S. Wei Y. Liu S. Peng H. Li Q. (2019). Silver nanoparticles exert concentration-dependent influences on biofilm development and architecture. Cell Prolif. 52, 1–8. 10.1111/cpr.12616 PMC6668980 31050052

[B63] Gutierrez A. M. Dziubla T. D. Hilt J. Z. (2017). Recent advances on iron oxide magnetic nanoparticles as sorbents of organic pollutants in water and wastewater treatment. Rev. Environ. Health 32, 111–117. 10.1515/reveh-2016-0063 28231068 PMC5785914

[B64] Haider A. J. Jameel Z. N. Al-Hussaini I. H. M. (2019). Review on: Titanium dioxide applications. Energy Procedia 157, 17–29. 10.1016/j.egypro.2018.11.159

[B65] Han J. Su H. Dong Q. Zhang D. Ma X. Zhang C. (2010). Patterning and photoluminescence of CdS nanocrystallites on silk fibroin fiber. J. Nanoparticle Res. 12, 347–356. 10.1007/s11051-009-9622-1

[B66] Heimbach B. Tonyali B. Zhang D. Wei M. (2018). High performance resorbable composites for load-bearing bone fixation devices. J. Mech. Behav. Biomed. Mater. 81, 1–9. 10.1016/j.jmbbm.2018.01.031 29471253

[B67] Hench L. L. (2009). Genetic design of bioactive glass. J. Eur. Ceram. Soc. 29, 1257–1265. 10.1016/j.jeurceramsoc.2008.08.002

[B68] Hoang Thi T. T. Lee Y. Le Thi P. Park K. D. (2019). Engineered horseradish peroxidase-catalyzed hydrogels with high tissue adhesiveness for biomedical applications. J. Ind. Eng. Chem. 78, 34–52. 10.1016/j.jiec.2019.05.026

[B69] Horváth T. Papp A. Igaz N. Kovács D. Kozma G. Trenka V. (2018). Pulmonary impact of titanium dioxide nanorods: examination of nanorod-exposed rat lungs and human alveolar cells. Int. J. Nanomedicine 13, 7061–7077. 10.2147/IJN.S179159 30464459 PMC6220432

[B70] Hotze E. M. Phenrat T. Lowry G. V. (2010). Nanoparticle aggregation: challenges to understanding transport and reactivity in the environment. J. Environ. Qual. 39, 1909–1924. 10.2134/jeq2009.0462 21284288

[B71] Hou X. Mu L. Chen F. Hu X. (2018). Emerging investigator series: design of hydrogel nanocomposites for the detection and removal of pollutants: from nanosheets, network structures, and biocompatibility to machine-learning-assisted design. Environ. Sci. Nano 5, 2216–2240. 10.1039/C8EN00552D

[B72] Hu C. Wu L. Zhou C. Sun H. Gao P. Xu X. (2020). Berberine/Ag nanoparticle embedded biomimetic calcium phosphate sca ff olds for enhancing antibacterial function. Nanotechnol. Rev. 9, 568–579. 10.1515/ntrev-2020-0046

[B73] Huang T. Fan C. Zhu M. Zhu Y. Zhang W. Li L. (2019). 3D-printed scaffolds of biomineralized hydroxyapatite nanocomposite on silk fibroin for improving bone regeneration. Appl. Surf. Sci. 467–468, 345–353. 10.1016/j.apsusc.2018.10.166

[B74] Iben Ayad A. Luart D. Ould Dris A. Guénin E. (2020). Kinetic analysis of 4-nitrophenol reduction by “water-soluble” palladium nanoparticles. Nanomaterials 10:1169 10.3390/nano10061169 PMC7353196 32549394

[B75] Ikawa T. Sajiki H. Hirota K. (2005). Highly chemoselective hydrogenation method using novel finely dispersed palladium catalyst on silk-fibroin: its preparation and activity. Tetrahedron 61, 2217–2231. 10.1016/j.tet.2004.11.080

[B76] James E. N. Van Doren E. Li C. Kaplan D. L. (2019). Silk biomaterials-mediated miRNA functionalized orthopedic devices. Tissue Eng. 25, 12–23. 10.1089/ten.tea.2017.0455 PMC6352554 29415631

[B77] Jayaram D. T. Runa S. Kemp M. L. Payne C. K. (2017). Nanoparticle-induced oxidation of corona proteins initiates an oxidative stress response in cells. Nanoscale 9, 7595–7601. 10.1039/C6NR09500C 28537609 PMC5703216

[B78] Jia M. Chen Z. Guo Y. Chen X. Zhao X. (2017). Efficacy of silk fibroin–nano silver against staphylococcus aureus biofilms in a rabbit model of sinusitis. Int. J. Nanomedicine 12, 2933–2939. 10.2147/IJN.S130160 28435269 PMC5391841

[B79] Jones J. R. (2013). Review of bioactive glass: from Hench to hybrids. Acta Biomater. 9, 4457–4486. 10.1016/j.actbio.2012.08.023 22922331

[B80] Jose R. R. Brown J. E. Polido K. E. Omenetto F. G. Kaplan D. L (2015). Polyol-silk bioink formulations as two-part room-temperature curable materials for 3D printing. ACS Biomater. Sci. Eng. 1, 780–788. 10.1021/acsbiomaterials.5b00160 33445255

[B81] Karageorgiou V. Kaplan D. (2005). Porosity of 3D biomaterial scaffolds and osteogenesis. Biomaterials 26, 5474–5491. 10.1016/j.biomaterials.2005.02.002 15860204

[B82] Karthikeyan K. Sekar S. Pandima Devi M. Inbasekaran S. Lakshminarasaiah C. H. Sastry T. P. (2011). Fabrication of novel biofibers by coating silk fibroin with chitosan impregnated with silver nanoparticles. J. Mater. Sci. Mater. Med. 22, 2721–2726. 10.1007/s10856-011-4462-9 22042460

[B83] Kim D. H. Viventi J. Amsden J. J. Xiao J. Vigeland L. Kim Y. S. (2010). Dissolvable films of silk fibroin for ultrathin conformal bio-integrated electronics. Nat. Mater. 9, 1–7. 10.1038/nmat2745 20400953 PMC3034223

[B84] Kim J. Kim D. Joo O. Woo H. Min J. Mi B. (2016). International Journal of Biological Macromolecules Osteoinductive silk fibroin/titanium dioxide/hydroxyapatite hybrid scaffold for bone tissue engineering. Int. J. Biol. Macromol. 82, 160–167. 10.1016/j.ijbiomac.2015.08.001 26257379

[B85] Kim J. W. Ki C. S. Um I. C. Park Y. H. (2017). A facile fabrication method and the boosted adsorption and photodegradation activity of CuO nanoparticles synthesized using a silk fibroin template. J. Ind. Eng. Chem. 56, 335–341. 10.1016/j.jiec.2017.07.029

[B86] Kim K. J. Sung W. S. Suh B. K. Moon S. K. Choi J. S. Kim J. G. (2009). Antifungal activity and mode of action of silver nano-particles on *Candida albicans*. Biometals 22, 235–242. 10.1007/s10534-008-9159-2 18769871

[B87] Koh L. D. Cheng Y. Teng C. P. Khin Y. W. Loh X. J. Tee S. Y. (2015). Structures, mechanical properties and applications of silk fibroin materials. Prog. Polym. Sci. 46, 86–110. 10.1016/j.progpolymsci.2015.02.001

[B88] Kojic N. Pritchard E. M. Tao H. Brenckle M. A. Mondia J. P. Panilaitis B. (2012). Focal infection treatment using laser-mediated heating of injectable silk hydrogels with gold nanoparticles. Adv. Funct. Mater. 22, 3793–3798. 10.1002/adfm.201200382 24015118 PMC3760432

[B89] Kolosnjaj-Tabi J. Marangon I. Nicolas-Boluda A. Silva A. K. A. Gazeau F. (2017). Nanoparticle-based hyperthermia, a local treatment modulating the tumor extracellular matrix. Pharmacol. Res. 126, 123–137. 10.1016/j.phrs.2017.07.010 28720518

[B90] Kong X. D. Cui F. Z. Wang X. M. Zhang M. Zhang W. (2004). Silk fibroin regulated mineralization of hydroxyapatite nanocrystals. J. Cryst. Growth 270, 197–202. 10.1016/j.jcrysgro.2004.06.007

[B91] Kundu B. Rajkhowa R. Kundu S. C. Wang X. (2013). Silk fibroin biomaterials for tissue regenerations. Adv. Drug Deliv. Rev. 65, 457–470. 10.1016/j.addr.2012.09.043 23137786

[B92] Kweon H. Y. Lee K. G. Chae C. H. Balázsi C. Min S. K. Kim J. Y. (2011). Development of nano-hydroxyapatite graft with silk fibroin scaffold as a new bone substitute. J. Oral Maxillofac. Surg. 69, 1578–1586. 10.1016/j.joms.2010.07.062 21272978

[B93] Lammel A. S. Hu X. Park S. H. Kaplan D. L. Scheibel T. R. (2010). Controlling silk fibroin particle features for drug delivery. Biomaterials 31, 4583–4591. 10.1016/j.biomaterials.2010.02.024 20219241 PMC2846964

[B94] Lan Y. Li W. Jiao Y. Guo R. Zhang Y. Xue W. (2014). Therapeutic efficacy of antibiotic-loaded gelatin microsphere/silk fibroin scaffolds in infected full-thickness burns. Acta Biomater. 10, 3167–3176. 10.1016/j.actbio.2014.03.029 24704698

[B95] Lan Y. Lu Y. Ren Z. (2013). Mini review on photocatalysis of titanium dioxide nanoparticles and their solar applications. Nano Energy 2, 1031–1045. 10.1016/j.nanoen.2013.04.002

[B96] Lara H. H Ayala-Núñez N. V. del Turrent L. C. I. Padilla C. R. (2010a). Bactericidal effect of silver nanoparticles against multidrug-resistant bacteria. World J. Microbiol. Biotechnol. 26, 615–621. 10.1007/s11274-009-0211-3

[B97] Lara H. H. Ayala-Nuñez N. V. Ixtepan-Turrent L. Rodriguez-Padilla C. (2010b). Mode of antiviral action of silver nanoparticles against HIV-1. J. Nanobiotechnology 8, 1–10. 10.1186/1477-3155-8-1 20145735 PMC2818642

[B98] Lawrence B. D. Marchant J. K. Pindrus M. A. Omenetto F. G. Kaplan D. L. (2009). Silk film biomaterials for cornea tissue engineering. Biomaterials 30, 1299–1308. 10.1016/j.biomaterials.2008.11.018 19059642 PMC2670567

[B99] Li A. B. Kluge J. A. Guziewicz N. A. Omenetto F. G. Kaplan D. L. (2015). Silk-based stabilization of biomacromolecules. J. Control. Release 219, 416–430. 10.1016/j.jconrel.2015.09.037 26403801 PMC4656123

[B100] Li C. Hotz B. Ling S. Guo J. Haas D. S. Marelli B. (2016). Regenerated silk materials for functionalized silk orthopedic devices by mimicking natural processing. Biomaterials 110, 24–33. 10.1016/j.biomaterials.2016.09.014 27697669 PMC5104183

[B101] Li G. Li Y. Chen G. He J. Han Y. Wang X. (2015). Silk-Based Biomaterials in Biomedical Textiles and Fiber-Based Implants. Adv. Healthc. Mater. 4, 1134–1151. 10.1002/adhm.201500002 25772248 PMC4456268

[B102] Li H. Zhu J. Chen S. Jia L. Ma Y. (2017). Fabrication of aqueous-based dual drug loaded silk fibroin electrospun nanofibers embedded with curcumin-loaded RSF nanospheres for drugs controlled release. RSC Adv. 7, 56550–56558. 10.1039/C7RA12394A

[B103] Li X. Liu Y. Zhang J. You R. Qu J. Li M. (2017). Functionalized silk fibroin dressing with topical bioactive insulin release for accelerated chronic wound healing. Mater. Sci. Eng. C 72, 394–404. 10.1016/j.msec.2016.11.085 28024602

[B104] Li Z. Song J. Zhang J. Hao K. Liu L. Wu B. (2020). Topical application of silk fibroin-based hydrogel in preventing hypertrophic scars. Coll. Surf. B Biointerf. 186:110735 10.1016/j.colsurfb.2019.110735 31865120

[B105] Liang B. Yu K. Ling Y. Kolios M. Exner A. Wang Z. (2019). An artificially engineered “tumor bio-magnet” for collecting blood-circulating nanoparticles and magnetic hyperthermia. Biomater. Sci. 7, 1815–1824. 10.1039/C8BM01658E 30916668

[B106] Ling S. Qin Z. Huang W. Cao S. Kaplan D. L. Buehler M. J. (2017). Design and function of biomimetic multilayer water purification membranes. Sci. Adv. 3, 1–12. 10.1126/sciadv.1601939 PMC5381955 28435877

[B107] Liu H. Wang Z. Li H. Wang H. Yu R. (2018). Controlled synthesis of silkworm cocoon-like α-Fe_2_O_3_ and its adsorptive properties for organic dyes and Cr(VI). Mater. Res. Bull. 100, 302–307. 10.1016/j.materresbull.2017.12.030

[B108] Liu J. Zhou J. Tang B. Zeng T. Li Y. Li J. (2016). Surface enhanced Raman scattering (SERS) fabrics for trace analysis. Appl. Surf. Sci. 386, 296–302. 10.1016/j.apsusc.2016.05.150

[B109] Lovett M. Cannizzaro C. Daheron L. Messmer B. Vunjak-Novakovic G. Kaplan D. L. (2007). Silk fibroin microtubes for blood vessel engineering. Biomaterials 28, 5271–5279. 10.1016/j.biomaterials.2007.08.008 17727944 PMC2695960

[B110] Lovett M. L. Wang X. Yucel T. York L. Keirstead M. Haggerty L. (2015). Silk hydrogels for sustained ocular delivery of anti-vascular endothelial growth factor (anti-VEGF) therapeutics. Eur. J. Pharm. Biopharm. 95, 271–278. 10.1016/j.ejpb.2014.12.029 25592326

[B111] Lu S. Wang X. Lu Q. Zhang X. Kluge J. A. Uppal N. (2010). Insoluble and flexible silk films containing glycerol. Biomacromolecules 11, 143–150. 10.1021/bm900993n 19919091

[B112] Lu Z. Mao C. Meng M. Liu S. Tian Y. Yu L. (2014a). Fabrication of CeO_2_ nanoparticle-modified silk for UV protection and antibacterial applications. J. Colloid Interface Sci. 435, 8–14. 10.1016/j.jcis.2014.08.015 25203972

[B113] Lu Z. Meng M. Jiang Y. Xie J. (2014b). UV-assisted *in situ* synthesis of silver nanoparticles on silk fibers for antibacterial applications. Colloids Surfaces A Physicochem. Eng. Asp. 447, 1–7. 10.1016/j.colsurfa.2014.01.064

[B114] Luo K.-y. Shao Z.-z. (2017). A novel regenerated silk fibroin-based hydrogels with magnetic and catalytic activities. Chinese J. Polym. Sci. 35, 515–523. 10.1007/s10118-017-1910-0

[B115] Ma R. Tsukruk V. V. (2017). Seriography-guided reduction of graphene oxide biopapers for wearable sensory electronics. Adv. Funct. Mater. 27:e201604802 10.1002/adfm.201604802

[B116] Maleki H. Huesing N. (2019). Silica-silk fibroin hybrid (bio)aerogels: two-step versus one-step hybridization. J. Sol-Gel Sci. Technol. 10.1007/s10971-019-04933-4 PMC8550194 34720431

[B117] Maleki H. Montes S. Hayati-Roodbari N. Putz F. Huesing N. (2018). Compressible, thermally insulating, and fire retardant aerogels through self-assembling silk fibroin biopolymers inside a silica structure - an approach towards 3D printing of aerogels. ACS Appl. Mater. Interfaces 10, 22718–22730. 10.1021/acsami.8b05856 29864277 PMC6513757

[B118] Mandal B. B. Kundu S. C. (2009). Cell proliferation and migration in silk fibroin 3D scaffolds. Biomaterials 30, 2956–2965. 10.1016/j.biomaterials.2009.02.006 19249094

[B119] Martínez-Prieto L. M. Cano I. Márquez A. Baquero E. A. Tricard S. Cusinato L. (2017). Zwitterionic amidinates as effective ligands for platinum nanoparticle hydrogenation catalysts. Chem. Sci. 8, 2931–2941. 10.1039/C6SC05551F 28451359 PMC5376718

[B120] Meddahi-Pellé A. Legrand A. Marcellan A. Louedec L. Letourneur D. Leibler L. (2014). Organ repair, hemostasis, and *in vivo* bonding of medical devices by aqueous solutions of nanoparticles. Angew. Chemie Int. Ed. 53, 6369–6373. 10.1002/anie.201401043 PMC4320763 24740730

[B121] Meng M. He H. Xiao J. Zhao P. Xie J. Lu Z. (2016). Controllable *in situ* synthesis of silver nanoparticles on multilayered film-coated silk fibers for antibacterial application. J. Colloid Interface Sci. 461, 369–375. 10.1016/j.jcis.2015.09.038 26414419

[B122] Midha S. Kumar S. Sharma A. Kaur K. Shi X. Naruphontjirakul P. (2018). Silk fibroin-bioactive glass based advanced biomaterials: towards patient-specific bone grafts. Biomed. Mater. 13:055012 10.1088/1748-605X/aad2a9 29995642

[B123] Mieszawska A. J. Fourligas N. Georgakoudi I. Ouhib N. M. Belton D. J. Perry C. C. (2010). Osteoinductive silk-silica composite biomaterials for bone regeneration. Biomaterials 31, 8902–8910. 10.1016/j.biomaterials.2010.07.109 20817293 PMC2949442

[B124] Monopoli M. P. Åberg C. Salvati A. Dawson K. A. (2012). Biomolecular coronas provide the biological identity of nanosized materials. Nat. Nanotechnol. 7, 779–786. 10.1038/nnano.2012.207 23212421

[B125] Mu X. Wang Y. Guo C. Li Y. Ling S. Huang W. (2020). 3D printing of silk protein structures by aqueous solvent-directed molecular assembly. Macromol. Biosci. 20:1900191 10.1002/mabi.201900191 PMC6980242 31433126

[B126] Nezhad-Mokhtari P. Ghorbani M. Roshangar L. Soleimani Rad J. (2019). Chemical gelling of hydrogels-based biological macromolecules for tissue engineering: Photo- and enzymatic-crosslinking methods. Int. J. Biol. Macromol. 139, 760–772. 10.1016/j.ijbiomac.2019.08.047 31400425

[B127] Ni W. Li M. Cui J. Xing Z. Li Z. Wu X. (2017). 808 nm light triggered black TiO2 nanoparticles for killing of bladder cancer cells. Mater. Sci. Eng. C 81, 252–260. 10.1016/j.msec.2017.08.020 28887971

[B128] Niu C. Li X. Wang Y. Liu X. Shi J. Wang X. (2019). Design and performance of a poly(vinyl alcohol)/silk fibroin enzymatically crosslinked semi-interpenetrating hydrogel for a potential hydrophobic drug delivery. RSC Adv. 9, 41074–41082. 10.1039/C9RA09344C PMC9076402 35540084

[B129] Ornell K. J. Taylor J. S. Zeki J. Ikegaki N. Shimada H. Coburn J. M. (2020). Local delivery of dinutuximab from lyophilized silk fibroin foams for treatment of an orthotopic neuroblastoma model. Cancer Med. 9, 2891–2903. 10.1002/cam4.2936 32096344 PMC7163090

[B130] Pandey S. Bodas D. (2020). High-quality quantum dots for multiplexed bioimaging: a critical review. Adv. Colloid Interface Sci. 278:102137 10.1016/j.cis.2020.102137 32171116

[B131] Partlow B. P. Hanna C. W. Rnjak-Kovacina J. Moreau J. E. Applegate M. B Burke K. A. (2014). Highly tunable elastomeric silk biomaterials. Adv. Funct. Mater. 24, 4615–4624. 10.1002/adfm.201400526 25395921 PMC4225629

[B132] Patil P. P. Meshram J. V. Bohara R. A. Nanaware S. G. Pawar S. H. (2018). ZnO nanoparticle-embedded silk fibroin-polyvinyl alcohol composite film: a potential dressing material for infected wounds. New J. Chem. 42, 14620–14629. 10.1039/C8NJ01675E

[B133] Patil S. George T. Mahadik K. (2015). Green synthesized nanosilver loaded silk fibroin gel for enhanced wound healing. J. Drug Deliv. Sci. Technol. 30, 30–36. 10.1016/j.jddst.2015.09.001

[B134] Pedone D. Moglianetti M. De Luca E. Bardi G. Pompa P. P. (2017). Platinum nanoparticles in nanobiomedicine. Chem. Soc. Rev. 46, 4951–4975. 10.1039/C7CS00152E 28696452

[B135] Phillips D. M. Drummy L. F. Conrady D. G. Fox D. M. Naik R. R. Stone M. O. (2004). Dissolution and regeneration of *Bombyx mori* silk fibroin using ionic liquids. J. Am. Chem. Soc. 126, 14350–14351. 10.1021/ja046079f 15521743

[B136] Pires F. Munhoz F. Koch L. M. Tanaka M. Souza M. Israelita H. (2019). Consenso Brasileiro de Nutrição em Transplante de Células-Tronco Hematopoiéticas : Idosos. Stem Cell Transplant. Elderly 17, 1–16. 10.31744/einstein_journal/2019AE4340

[B137] Plan Sangnier A. Aufaure R. Cheong S. Motte L. Palpant B. Tilley R. D. (2019). Raspberry-like small multicore gold nanostructures for efficient photothermal conversion in the first and second near-infrared windows. Chem. Commun. 55, 4055–4058. 10.1039/C8CC09476D 30875417

[B138] Plan Sangnier A. Van de Walle A. Aufaure R. Fradet M. Motte L. Guénin E. (2020). Endosomal confinement of gold nanospheres, nanorods, and nanoraspberries governs their photothermal identity and is beneficial for cancer cell therapy. Adv. Biosyst. 4:1900284 10.1002/adbi.201900284 32293165

[B139] Poza P. Pérez-Rigueiro J. Elices M. Lorca J. (2002). Fractographic analysis of silkworm and spider silk. Eng. Fract. Mech. 69, 1035–1048. 10.1016/S0013-7944(01)00120-5

[B140] Qian K.-Y. Song Y. Yan X. Dong L. Xue J. Xu Y. (2020). Injectable ferrimagnetic silk fibroin hydrogel for magnetic hyperthermia ablation of deep tumor. Biomaterials 259:120299 10.1016/j.biomaterials.2020.120299 32827797

[B141] Qian L. Zhang H. (2011). Controlled freezing and freeze drying: a versatile route for porous and micro-/nano-structured materials. J. Chem. Technol. Biotechnol. 86, 172–184. 10.1002/jctb.2495

[B142] Raho R. Nguyen N. Y. Zhang N. Jiang W. Sannino A. Liu H. (2020). Photo-assisted green synthesis of silver doped silk fibroin/carboxymethyl cellulose nanocomposite hydrogels for biomedical applications. Mater. Sci. Eng. C 107:110219 10.1016/j.msec.2019.110219 31761177

[B143] Raja N. Yun H. S. (2016). A simultaneous 3D printing process for the fabrication of bioceramic and cell-laden hydrogel core/shell scaffolds with potential application in bone tissue regeneration. J. Mater. Chem. B 4, 4707–4716. 10.1039/C6TB00849F 32263243

[B144] Ranjana R. Parushuram N. Harisha K. S. Asha S. Sangappa Y. (2020). Silk fibroin a bio-template for synthesis of different shaped gold nanoparticles: characterization and ammonia detection application. Mater. Today Proc. 27, 434–439. 10.1016/j.matpr.2019.11.259

[B145] Rao V. N. Reddy N. L. Kumari M. M. Cheralathan K. K. Ravi P. Sathish M. (2019). Sustainable hydrogen production for the greener environment by quantum dots-based efficient photocatalysts: a review. J. Environ. Manage. 248:109246 10.1016/j.jenvman.2019.07.017 31323456

[B146] Reidy B. Haase A. Luch A. Dawson K. A. Lynch I. (2013). Mechanisms of silver nanoparticle release, transformation and toxicity: a critical review of current knowledge and recommendations for future studies and applications. Materials 6, 2295–2350. 10.3390/ma6062295 28809275 PMC5458943

[B147] Ribeiro M. De Moraes M. A. Beppu M. M. Garcia M. P. Fernandes M. H. Monteiro F. J. (2015). Development of silk fibroin/nanohydroxyapatite composite hydrogels for bone tissue engineering. Eur. Polym. J. 67, 66–77. 10.1016/j.eurpolymj.2015.03.056

[B148] Ribeiro M. Feraz M. P. Monteiro F. J. Fernandes M. H. Beppu M. M. Mantione D. (2017). Antibacterial silk fibroin/nanohydroxyapatite hydrogels with silver and gold nanoparticles for bone regeneration. Nanomed. Nanotechnol. Biol. Med. 13, 231–239. 10.1016/j.nano.2016.08.026 27591960

[B149] Rockwood D. N. Preda R. C. Yücel T. Wang X. Lovett M. L. and Kaplan D. L. (2011). Materials fabrication from *Bombyx mori* silk fibroin. Nat. Protoc. 6, 1612–1631. 10.1038/nprot.2011.379 21959241 PMC3808976

[B150] Rodriguez M. J. Dixon T. A. Cohen E. Huang W. Omenetto F. G. and Kaplan D. L. (2018). 3D freeform printing of silk fibroin. Acta Biomater. 71, 379–387. 10.1016/j.actbio.2018.02.035 29550442 PMC5899947

[B151] Runa S. Lakadamyali M. Kemp M. L. Payne C. K. (2017). TiO_2_ nanoparticle-induced oxidation of the plasma membrane: importance of the protein corona. J. Phys. Chem. B 121, 8619–8625. 10.1021/acs.jpcb.7b04208 28844138

[B152] Saldan I. Semenyuk Y. Marchuk I. Reshetnyak O. (2015). Chemical synthesis and application of palladium nanoparticles. J. Mater. Sci. 50, 2337–2354. 10.1007/s10853-014-8802-2

[B153] Samadi A. Klingberg H. Jauffred L. Kjær A. Bendix P. M. Oddershede L. B. (2018). Platinum nanoparticles: a non-toxic, effective and thermally stable alternative plasmonic material for cancer therapy and bioengineering. Nanoscale 10, 9097–9107. 10.1039/C8NR02275E 29718060

[B154] Santos L. J. Reis R. L. Gomes M. E. (2015). Harnessing magnetic-mechano actuation in regenerative medicine and tissue engineering. Trends Biotechnol. 33, 471–479. 10.1016/j.tibtech.2015.06.006 26123708

[B155] Schmucker A. L. Dickerson M. B. Rycenga M. Mangelson B. F. Brown K. A. Naik R. R. (2014). Combined chemical and physical encoding with silk fibroin-embedded nanostructures. Small 10, 1485–1489. 10.1002/smll.201302923 24376130

[B156] Shahid M. Cheng X. W. Tang R. C. Chen G. (2017). Silk functionalization by caffeic acid assisted *in-situ* generation of silver nanoparticles. Dye. Pigment. 137, 277–283. 10.1016/j.dyepig.2016.10.009

[B157] Sheikh F. A. Ju H. W. Lee J. M. Moon B. M. Park H. J. Lee O. J. (2015). 3D electrospun silk fibroin nanofibers for fabrication of artificial skin. Nanomed. Nanotechnol. Biol. Med. 11, 681–691. 10.1016/j.nano.2014.11.007 25555351

[B158] Smith A. T. LaChance A. M. Zeng S. Liu B. Sun L. (2019). Synthesis, properties, and applications of graphene oxide/reduced graphene oxide and their nanocomposites. Nano Mater. Sci. 1, 31–47. 10.1016/j.nanoms.2019.02.004

[B159] Sohail Haroone M. Li L. Ahmad A. Huang Y. Ma R. Zhang P. (2018). Luminous composite ultrathin films of CdTe quantum dots/silk fibroin co-assembled with layered doubled hydroxide: enhanced photoluminescence and biosensor application. J. Mater. 4, 165–171. 10.1016/j.jmat.2018.05.002

[B160] Sohail A. Ahmad Z. Bég O. A. Arshad S. Sherin L. (2017). Revue sur le traitement par hyperthermie médiée par nanoparticules. Bull. Cancer 104, 452–461. 10.1016/j.bulcan.2017.02.003 28385267

[B161] Sommer M. R. Schaffner M. Carnelli D. Studart A. R. (2016). 3D Printing of hierarchical silk fibroin structures. ACS Appl. Mater. Interfaces 8, 34677–34685. 10.1021/acsami.6b11440 27933765

[B162] Song J. Zhang P. Cheng L. Liao Y. Xu B. Bao R. (2015). Nano-silver *in situ* hybridized collagen scaffolds for regeneration of infected full-thickness burn skin. J. Mater. Chem. B 3, 4231–4241. 10.1039/C5TB00205B 32262300

[B163] Song W. Muthana M. Mukherjee J. Falconer R. J. Biggs C. A. Zhao X. (2017). Magnetic-silk core–shell nanoparticles as potential carriers for targeted delivery of curcumin into human breast cancer cells. ACS Biomater. Sci. Eng. 3, 1027–1038. 10.1021/acsbiomaterials.7b00153 33429579

[B164] Sridhar S. Venugopal J. R. Sridhar R. Ramakrishna S. (2015). Cardiogenic differentiation of mesenchymal stem cells with gold nanoparticle loaded functionalized nanofibers. Coll. Surf. B Biointerf. 134, 346–354. 10.1016/j.colsurfb.2015.07.019 26209968

[B165] Stankic S. Suman S. Haque F. Vidic J. (2016). Pure and multi metal oxide nanoparticles: synthesis, antibacterial and cytotoxic properties. J. Nanobiotechnol. 14, 1–20. 10.1186/s12951-016-0225-6 PMC5075760 27776555

[B166] Stinson J. A. Palmer C. R. Miller D. P. Li A. B. Lightner K. Jost H. (2020). Thin silk fibroin films as a dried format for temperature stabilization of inactivated polio vaccine. Vaccine 38, 1652–1660. 10.1016/j.vaccine.2019.12.062 31959422 PMC7176408

[B167] Su S. S. Chang I. (2017). “Review of production routes of nanomaterials,” in Commercialization of Nanotechnologies-A Case Study Approach, eds D. Brabazon, E. Pellicer, F. Zivic, J. Sort, M. Dolors Baró, N. Grujovic, et al. (Cham: Springer International Publishing), 15–29.

[B168] Sun J. Shen Q. Y. Lu J. X. (2009). Comparative study of microstructural remodification to porous β-TCP and HA in rabbits. Chinese Sci. Bull. 54, 2962–2967. 10.1007/s11434-009-0332-y

[B169] Sun L. Parker S. T. Syoji D. Wang X. Lewis J. A. Kaplan D. L. (2012). Direct-write assembly of 3D silk/hydroxyapatite scaffolds for bone co-cultures. Adv. Healthc. Mater. 1, 729–735. 10.1002/adhm.201200057 23184824 PMC3739986

[B170] Suzuki T. Miura N. Hojo R. Yanagiba Y. Suda M. Hasegawa T. (2020). Genotoxicity assessment of titanium dioxide nanoparticle accumulation of 90 days in the liver of gpt delta transgenic mice. Genes Environ. 42:7 10.1186/s41021-020-00151-5 32071618 PMC7011542

[B171] Szekeres G. P. Kneipp J. (2019). SERS probing of proteins in gold nanoparticle agglomerates. Front. Chem. 7, 1–10. 10.3389/fchem.2019.00030 30766868 PMC6365451

[B172] Tanasa E. Zaharia C. Hudita A. Radu I. C. Costache M. Galateanu B. (2020). Impact of the magnetic field on 3T3-E1 preosteoblasts inside SMART silk fibroin-based scaffolds decorated with magnetic nanoparticles. Mater. Sci. Eng. C 110, 1–13. 10.1016/j.msec.2020.110714 32204026

[B173] Tang B. Sun L. Kaur J. Yu Y. Wang X. (2014). *In-situ* synthesis of gold nanoparticles for multifunctionalization of silk fabrics. Dye. Pigment. 103, 183–190. 10.1016/j.dyepig.2013.12.008

[B174] Tao H. Siebert S. M. Brenckle M. A. Averitt R. D. Cronin-Golomb M. Kaplan D. L. (2010). Gold nanoparticle-doped biocompatible silk films as a path to implantable thermo-electrically wireless powering devices. Appl. Phys. Lett. 97:123702 10.1063/1.3486157

[B175] Thebo K. H. Qian X. Zhang Q. Chen L. Cheng H. M. Ren W. (2018). Highly stable graphene-oxide-based membranes with superior permeability. Nat. Commun. 9, 1–8. 10.1038/s41467-018-03919-0 29662053 PMC5902455

[B176] Tsioris K. Raja W. K. Pritchard E. M. Panilaitis B. Kaplan D. L. Omenetto F. G. (2012). Fabrication of silk microneedles for controlled-release drug delivery. Adv. Funct. Mater. 22, 330–335. 10.1002/adfm.201102012

[B177] Uttayarat P. Jetawattana S. Suwanmala P. Eamsiri J. Tangthong T. Pongpat S. (2012). Antimicrobial electrospun silk fibroin mats with silver nanoparticles for wound dressing application. Fibers Polym. 13, 999–1006. 10.1007/s12221-012-0999-6

[B178] Van de Walle A. Sangnier A. P. Abou-Hassan A. Curcio A. Hémadi M. Menguy N. (2019). Biosynthesis of magnetic nanoparticles from nanodegradation products revealed in human stem cells. Proc. Natl. Acad. Sci. U.S.A. 116, 4044–4053. 10.1073/pnas.1816792116 30760598 PMC6410821

[B179] Van Der Sanden B. Dhobb M. Berger F. Wion D. (2010). Optimizing stem cell culture. J. Cell. Biochem. 111, 801–807. 10.1002/jcb.22847 20803548 PMC3348118

[B180] van Loo B. Salehi S. S. Henke S. Shamloo A. Kamperman T. Karperien M. (2020). Enzymatic outside-in crosslinking enables single-step microcapsule production for high-throughput 3D cell microaggregate formation. Mater. Today Bio. 6:100047 10.1016/j.mtbio.2020.100047 PMC7152680 32300754

[B181] Velmurugan P. Shim J. Kim H. W. Lim J. M. Kim S. A. Seo Y. S. (2020). Bio-functionalization of cotton, silk, and leather using different *in-situ* silver nanoparticle synthesis modules, and their antibacterial properties. Res. Chem. Intermed. 46, 999–1015. 10.1007/s11164-016-2481-3

[B182] Vepari C. Kaplan D. L. (2007). Silk as a biomaterial. Prog. Polym. Sci. 32, 991–1007. 10.1016/j.progpolymsci.2007.05.013 19543442 PMC2699289

[B183] Vidal S. E. L. Tamamoto K. A. Nguyen H. Abbott R. D. Cairns D. M. Kaplan D. L. (2019). 3D biomaterial matrix to support long term, full thickness, immuno-competent human skin equivalents with nervous system components. Biomaterials 198, 194–203. 10.1016/j.biomaterials.2018.04.044 29709325 PMC6200656

[B184] Wang F. Jyothirmayee Aravind S. S. Wu H. Forys J. Venkataraman V. Ramanujachary K. (2017). Tunable green graphene-silk biomaterials: mechanism of protein-based nanocomposites. Mater. Sci. Eng. C 79, 728–739. 10.1016/j.msec.2017.05.120 28629074

[B185] Wang J. Zhang Y. Jin N. Mao C. Yang M. (2019). Protein-induced gold nanoparticle assembly for improving the photothermal effect in cancer therapy. ACS Appl. Mater. Interfaces 11, 11136–11143. 10.1021/acsami.8b21488 30869510

[B186] Wang L. Lu R. Hou J. Nan X. Xia Y. Guo Y. (2020). Application of injectable silk fibroin/graphene oxide hydrogel combined with bone marrow mesenchymal stem cells in bone tissue engineering. Coll. Surf. A Physicochem. Eng. Asp. 604:125318 10.1016/j.colsurfa.2020.125318

[B187] Wang S. Zhu M. Zhao L. Kuang D. Kundu S. C. Lu S. (2019). Insulin-loaded silk fibroin microneedles as sustained release system. ACS Biomater. Sci. Eng. 5, 1887–1894. 10.1021/acsbiomaterials.9b00229 33405562

[B188] Watanabe K. Miwa E. Asai F. Seki T. Urayama K. Nakatani T. (2020). Highly transparent and tough filler composite elastomer inspired by the cornea. ACS Mater. Lett. 2, 325–330. 10.1021/acsmaterialslett.9b00520

[B189] Wei G. Ma P. X. (2004). Structure and properties of nano-hydroxyapatite/polymer composite scaffolds for bone tissue engineering. Biomaterials 25, 4749–4757. 10.1016/j.biomaterials.2003.12.005 15120521

[B190] Wu J. Zheng K. Huang X. Liu J. Liu H. Boccaccini A. R. (2019). Thermally triggered injectable chitosan/silk fibroin/bioactive glass nanoparticle hydrogels for *in-situ* bone formation in rat calvarial bone defects. Acta Biomater. 91, 60–71. 10.1016/j.actbio.2019.04.023 30986530

[B191] Wu W. Jiang C. Z. Roy V. A. L. (2016). Designed synthesis and surface engineering strategies of magnetic iron oxide nanoparticles for biomedical applications. Nanoscale 8, 19421–19474. 10.1039/C6NR07542H 27812592

[B192] Xiong R. Grant A. M. Ma R. Zhang S. Tsukruk V. V. (2018). Naturally-derived biopolymer nanocomposites: interfacial design, properties and emerging applications. Mater. Sci. Eng. R Reports 125, 1–41. 10.1016/j.mser.2018.01.002

[B193] Xu Z. Shi L. Yang M. Zhu L. (2019). Preparation and biomedical applications of silk fibroin-nanoparticles composites with enhanced properties - a review. Mater. Sci. Eng. C 95, 302–311. 10.1016/j.msec.2018.11.010 30573254

[B194] Yang G. Lin H. Rothrauff B. B. Yu S. Tuan R. S. (2016). Multilayered polycaprolactone/gelatin fiber-hydrogel composite for tendon tissue engineering. Acta Biomater. 35, 68–76. 10.1016/j.actbio.2016.03.004 26945631 PMC5408748

[B195] Yang Y. Chen M. Wu Y. Wang P. Zhao Y. Zhu W. (2019). Ultrasound assisted one-step synthesis of Au@Pt dendritic nanoparticles with enhanced NIR absorption for photothermal cancer therapy. RSC Adv. 9, 28541–28547. 10.1039/C9RA04286E PMC9071120 35529621

[B196] Ye P. Yu B. Deng J. She R. F. Huang W. L. (2017). Application of silk fibroin/chitosan/nano-hydroxyapatite composite scaffold in the repair of rabbit radial bone defect. Exp. Ther. Med. 14, 5547–5553. 10.3892/etm.2017.5231 29285090 PMC5740707

[B197] Yin H. Ai S. Shi W. Zhu L. (2009a). A novel hydrogen peroxide biosensor based on horseradish peroxidase immobilized on gold nanoparticles-silk fibroin modified glassy carbon electrode and direct electrochemistry of horseradish peroxidase. Sens. Actuat. B Chem. 137, 747–753. 10.1016/j.snb.2008.12.046

[B198] Yin H. Ai S. Xu J. Shi W. Zhu L. (2009b). Amperometric biosensor based on immobilized acetylcholinesterase on gold nanoparticles and silk fibroin modified platinum electrode for detection of methyl paraoxon, carbofuran and phoxim. J. Electroanal. Chem. 637, 21–27. 10.1016/j.jelechem.2009.09.025

[B199] Yin Z. Kuang D. Wang S. Zheng Z. Yadavalli V. K. Lu S. (2018). Swellable silk fibroin microneedles for transdermal drug delivery. Int. J. Biol. Macromol. 106, 48–56. 10.1016/j.ijbiomac.2017.07.178 28778522

[B200] Yu K. Lu F. Li Q. Chen H. Lu B. Liu J. (2017). *In situ* assembly of Ag nanoparticles (AgNPs) on porous silkworm cocoon-based would film: enhanced antimicrobial and wound healing activity. Sci. Rep. 7, 1–13. 10.1038/s41598-017-02270-6 28522813 PMC5437089

[B201] Yu W. Kuzuya T. Hirai S. Tamada Y. Sawada K. Iwasa T. (2012). Preparation of Ag nanoparticle dispersed silk fibroin compact. Appl. Surf. Sci. 262, 212–217. 10.1016/j.apsusc.2012.05.084

[B202] Yukseloglu S. M. Sokmen N. Canoglu S. (2015). Biomaterial applications of silk fibroin electrospun nanofibres. Microelectron. Eng. 146, 43–47. 10.1016/j.mee.2015.04.008

[B203] Zakharov N. A. Demina L. I. Aliev A. D. Kiselev M. R. Matveev V. V. Orlov M. A. (2017). Synthesis and properties of calcium hydroxyapatite/silk fibroin organomineral composites. Inorg. Mater. 53, 333–342. 10.1134/S0020168517030128

[B204] Zhang G. Liu Y. Gao X. Chen Y. (2014). Synthesis of silver nanoparticles and antibacterial property of silk fabrics treated by silver nanoparticles. Nanoscale Res. Lett. 9, 1–8. 10.1186/1556-276X-9-216 24872803 PMC4022402

[B205] Zhang L. Liu X. Li G. Wang P. Yang Y. (2019). Tailoring degradation rates of silk fibroin scaffolds for tissue engineering. J. Biomed. Mater. Res. Part A 107, 104–113. 10.1002/jbm.a.36537 30367546

[B206] Zhang S. Zhou Z. Zhong J. Shi Z. Mao Y. Tao T. H. (2020). Body-integrated, enzyme-triggered degradable, silk-based mechanical sensors for customized health/fitness monitoring and *in situ* treatment. Adv. Sci. 7, 1–10. 10.1002/advs.202070071 PMC7341100 32670755

[B207] Zhang W. Chen L. Chen J. Wang L. Gui X. Ran J. (2017). Silk fibroin biomaterial shows safe and effective wound healing in animal models and a randomized controlled clinical trial. Adv. Healthc. Mater. 6, 1–16. 10.1002/adhm.201700121 28337854

[B208] Zheng Z. Wu J. Liu M. Wang H. Li C. Rodriguez M. J. (2018). 3D Bioprinting of self-standing silk-based bioink. Adv. Healthc. Mater. 7, 1–12. 10.1002/adhm.201701026 29292585

[B209] Zhou H. Wang X. Wang T. Zeng J. Yuan Z. Jian J. (2019). *In situ* decoration of Ag@AgCl nanoparticles on polyurethane/silk fibroin composite porous films for photocatalytic and antibacterial applications. Eur. Polym. J. 118, 153–162. 10.1016/j.eurpolymj.2019.05.058

[B210] Zhou J. Zhang B. Shi L. Zhong J. Zhu J. Yan J. (2014). Regenerated silk fibroin films with controllable nanostructure size and secondary structure for drug delivery. ACS Appl. Mater. Interfaces 6, 21813–21821. 10.1021/am502278b 25536875

[B211] Zhou L. Yu K. Lu F. Lan G. Dai F. Shang S. (2020). Minimizing antibiotic dosage through *in situ* formation of gold nanoparticles across antibacterial wound dressings: a facile approach using silk fabric as the base substrate. J. Clean. Prod. 243:118604 10.1016/j.jclepro.2019.118604

[B212] Zhou W. Gao X. Liu D. Chen X. (2015). Gold nanoparticles for *in vitro* diagnostics. Chem. Rev. 115, 10575–10636. 10.1021/acs.chemrev.5b00100 26114396 PMC5226399

[B213] Zhou Y. Tang R. C. (2018a). Facile and eco-friendly fabrication of AgNPs coated silk for antibacterial and antioxidant textiles using honeysuckle extract. J. Photochem. Photobiol. B Biol. 178, 463–471. 10.1016/j.jphotobiol.2017.12.003 29223813

[B214] Zhou Y. Tang R. C. (2018b). Facile and eco-friendly fabrication of colored and bioactive silk materials using silver nanoparticles synthesized by two flavonoids. Polymers 10:404 10.3390/polym10040404 PMC6415457 30966439

[B215] Ziental D. Czarczynska-Goslinska B. Mlynarczyk D. T. Glowacka-Sobotta A. Stanisz B. Goslinski T. (2020). Titanium dioxide nanoparticles: prospects and applications in medicine. Nanomaterials 10:387 10.3390/nano10020387 PMC7075317 32102185

[B216] Zou F. Zhou J. Zhang J. Li J. Tang B. Chen W. (2018). Functionalization of silk with *in-situ* synthesized platinum nanoparticles. Materials 11, 1–13. 10.3390/ma11101929 PMC6213640 30309006

